# Spin Crossover and Long‐Lived Excited States in a Reduced Molecular Ruby

**DOI:** 10.1002/chem.202001237

**Published:** 2020-05-20

**Authors:** Patrick M. Becker, Christoph Förster, Luca M. Carrella, Pit Boden, David Hunger, Joris van Slageren, Markus Gerhards, Eva Rentschler, Katja Heinze

**Affiliations:** ^1^ Department of Chemistry Johannes Gutenberg University of Mainz Duesbergweg 10-14 55128 Mainz Germany; ^2^ Department of Chemistry and Research Center Optimas University Kaiserslautern Erwin-Schrödinger-Straße 67663 Kaiserslautern Germany; ^3^ Institute of Physical Chemistry and Center for, Integrated Quantum Science and Technology University of Stuttgart Pfaffenwaldring 55 70569 Stuttgart Germany

**Keywords:** chromium, excited states, magnetic properties, spin crossover, step-scan IR spectroscopy

## Abstract

The chromium(III) complex [Cr^III^(ddpd)_2_]^3+^ (molecular ruby; ddpd=*N*,*N′*‐dimethyl‐*N*,*N′*‐dipyridine‐2‐yl‐pyridine‐2,6‐diamine) is reduced to the genuine chromium(II) complex [Cr^II^(ddpd)_2_]^2+^ with d^4^ electron configuration. This reduced molecular ruby represents one of the very few chromium(II) complexes showing spin crossover (SCO). The reversible SCO is gradual with *T*_1/2_ around room temperature. The low‐spin and high‐spin chromium(II) isomers exhibit distinct spectroscopic and structural properties (UV/Vis/NIR, IR, EPR spectroscopies, single‐crystal XRD). Excitation of [Cr^II^(ddpd)_2_]^2+^ with UV light at 20 and 290 K generates electronically excited states with microsecond lifetimes. This initial study on the unique reduced molecular ruby paves the way for thermally and photochemically switchable magnetic systems based on chromium complexes complementing the well‐established iron(II) SCO systems.

## Graphical abstract

**Ruby‐reduced**: Reduction of the molecular ruby [Cr^III^(ddpd)_2_]^3+^ (ddpd=*N*,*N′*‐dimethyl‐*N*,*N′*‐dipyridine‐2‐yl‐pyridine‐2,6‐diamine) gives the genuine chromium(II) complex [Cr^II^(ddpd)_2_]^2+^ showing spin crossover (SCO) with *T*_1/2_ around room temperature. Geometric distortions and color changes accompany this reversible SCO. Excitation with UV light yields electronically excited states with microsecond lifetimes.

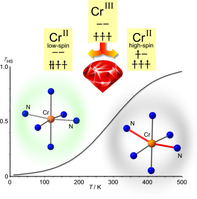

Spin crossover (SCO) in octahedral transition metal complexes can occur in the d^4^–d^7^ electron configurations.^1^ While systems with d^5^–d^7^ electron configurations (Fe^III^, Fe^II^, Co^II^) have been extensively studied and already matured towards applications,^2^, ^3^, ^4^, ^5^, ^6^, ^7^ the d^4^ SCO case (Cr^II^, Mn^III^) is only rarely observed and is underdeveloped.^8^, ^9^, ^10^, ^11^, ^12^, ^13^, ^14^ While several manganese(III) SCO complexes with d^4^ electron configuration have been reported,^15^, ^16^, ^17^, ^18^, ^19^, ^20^ the only three reported chromium(II) complexes close to the SCO point are *trans*‐CrI_2_(depe)_2_ (*T*_1/2_ = 170 K, depe = 1,2‐bis(diethylphosphano)ethane),^8^, ^9^, ^10^, ^11^, ^12^ Cp^*i*Pr4^CrCp^*i*Pr4^ (*T*_1/2_ ≈ 190 K, Cp^*i*Pr4^ = tetraisopropylcyclopentadienide),^13^ and Cp*Cr(η^5^‐P_5_)CrCp* with (*T*_1/2_ < 190 K, Cp* = pentamethylcyclopentadienide) (Scheme 1).^14^ Significantly, strong‐field ligands, such as phosphanes or cyclopentadienyl ligands coordinate to the Cr^II^ ion to allow for SCO. The very strong cyanido ligand yields the salts M_4_[Cr^II^(CN)_6_] (M=Na, K), which exhibit room temperature magnetic moments close to the expected spin‐only value for a low‐spin d^4^ complex.^21^, ^22^

**Scheme 1:**
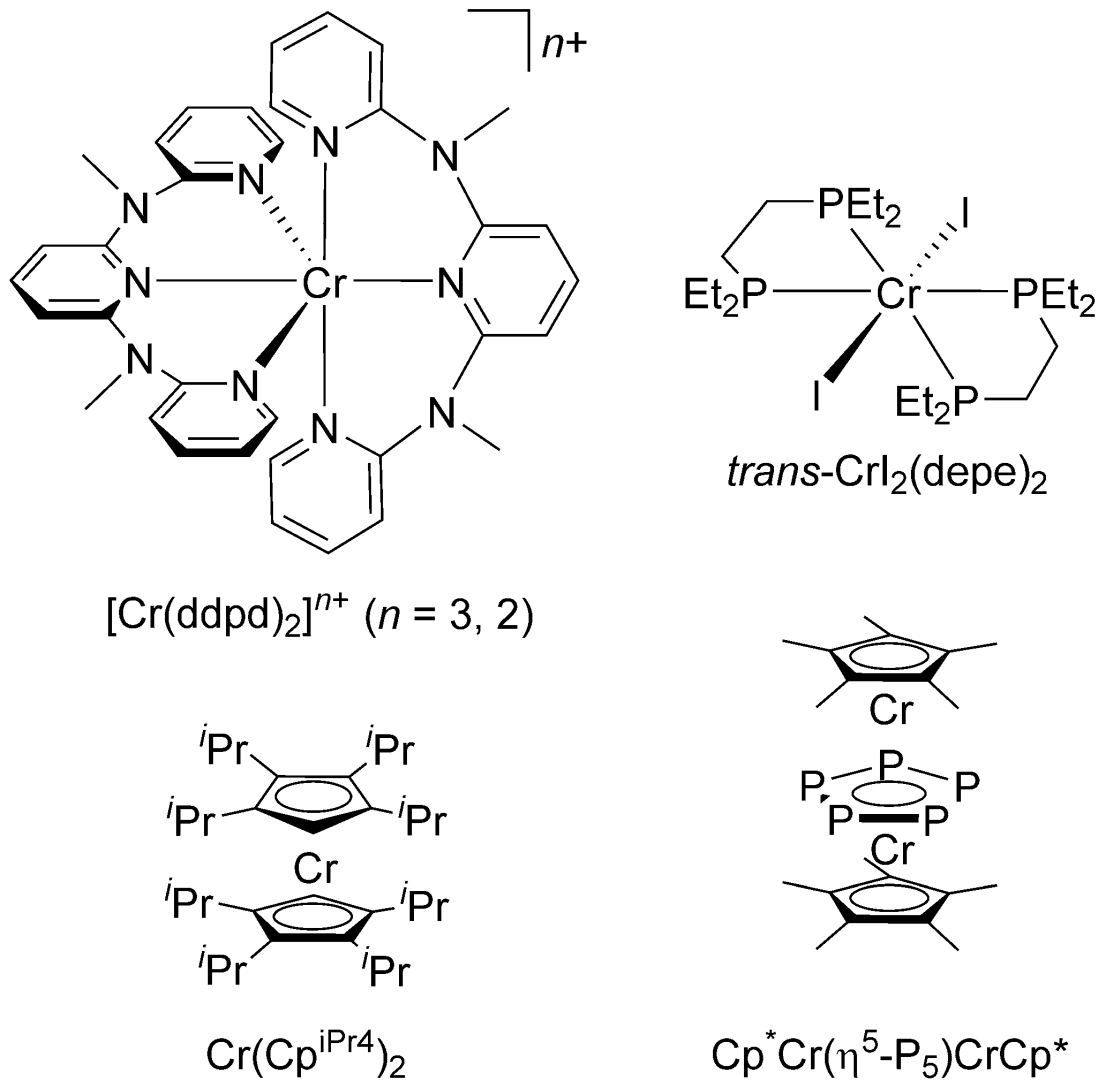
Molecular ruby and reduced molecular ruby [Cr(ddpd)_2_]^*n*+^ and the three chromium(II) SCO complexes reported so far.

The molecular ruby [Cr^III^(ddpd)_2_]^3+^ (d^3^ electron configuration, ddpd=*N*,*N′*‐dimethyl‐*N*,*N′*‐dipyridine‐2‐yl‐pyridine‐2,6‐diamine) shows exceptional photophysical properties, such as high photoluminescence quantum yield up to 30 % (as deuterated derivative) and high luminescence lifetimes of the ^2^T_1_/^2^E states (notation in octahedral symmetry).^23^, ^24^ These features led to applications in optical temperature and pressure sensing, circularly polarized luminescence, singlet oxygen formation and photocatalysis.^25^, ^26^, ^27^, ^28^, ^29^, ^30^, ^31^ Furthermore, the large ground state spin (*S=* ^3^/_2_; ^4^A_2_) of the chromium(III) complex enables the utilization as molecular quantum bit with phase memory times of 4.25 and 8.4 μs in protio and deuterio solvents at 7 K.^32^

One key to these properties lies in the strong‐field ligand ddpd forming six‐membered chelate rings. Photophysical and redox properties contrast those of the chromium(III) complexes [Cr(bpy)_3_]^3+^, [Cr(phen)_3_]^3+^, [Cr(tpy)_2_]^3+^ and [Cr(tpe)_2_]^3+^ featuring electron‐poor pyridine ligands (bpy=2,2′‐bipyridine, phen=1,10‐phenanthroline, tpy=2,2′:6′,2′′‐terpyridine, tpe=1,1,1‐tris(pyrid‐2‐yl)ethane).^33^, ^34^, ^35^, ^36^, ^37^, ^38^, ^39^ Consequently, the latter complexes show ligand‐centered redox chemistry yielding for example, [Cr^III^(tpy^⋅−^)(tpy)]^2+^, but no low‐spin chromium(II).^33^, ^34^ This ligand‐based redox chemistry also enables a rich photo‐redox chemistry.^35^, ^36^, ^37^, ^38^, ^39^ On the other hand, [Cr(ddpd)_2_]^3+^ gives the genuine chromium(II) complex [Cr(ddpd)_2_]^2+^ upon reduction.^40^ The magnetic susceptibility at room temperature (*χT*=2.67 cm^3^ K mol^−1^) is somewhat below that expected for a pure high‐spin complex (*χT*=3.00 cm^3^ K mol^−1^). In the solid state at 263 K the [CrN_6_] coordination polyhedron of [Cr(ddpd)_2_][BF_4_]_2_ **⋅**2 CH_3_CN shows a Jahn–Teller distortion^41^, ^42^ towards an elongated octahedron, similar to the Jahn–Teller ion [Cu(ddpd)_2_]^2+^.^40^, ^43^ Yet, the Cr−N distances are not very distinct at this temperature. All these data are consistent with a prevailing high‐spin electron configuration of the d^4^‐Cr^III^ ion at these high temperatures. On the other hand, the ddpd ligand is a strong‐field ligand, so we surmised that a low‐spin chromium(II) configuration, similar to [Cr(CN)_6_]^3−^,^21^, ^22^ could be accessible with this complex.

The *χT* product of a solid sample of [Cr(ddpd)_2_][BF_4_]_2_ gradually drops from 2.37 cm^3^ K mol^−1^ at 350 K to 1.01 cm^3^ K mol^−1^ at 50 K (Figure 1). We attribute this observation to SCO from high‐spin to low‐spin Cr^II^. Below ca. 20 K, *χT* further decreases due to zero‐field splitting (zfs). The low‐temperature data can be simulated with *g=*2.000(3) and a zfs of *D*=+5.95(12) cm^−1^ (Supporting Information, Figure S4 a). A negative *D* value failed to give a satisfactory simulation.

**Figure 1:**
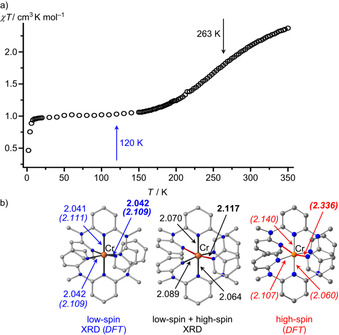
a) *χT* vs. *T* plot of [Cr(ddpd)_2_][BF_4_]_2_ at 0.1 T. b) Molecular geometries of the cations of [Cr(ddpd)_2_][BF_4_]_2_×2CH_3_CN at 120 K (low‐spin) and 263 K (low‐spin+high spin) and DFT calculated high‐spin structure; relevant distances in Å, Jahn–Teller axis indicated in red.

To substantiate the zero‐field splitting of the low‐spin (triplet) state, high‐field EPR spectra of [Cr(ddpd)_2_][BF_4_]_2_ were recorded at 5 K between 180 and 375 GHz (Supporting Information, Figure S5).^32^, ^44^ The simulation^45^ yielded the following spin Hamiltonian parameters *g_x_*=2.08(5), *g_y_*=2.10(5), *g_z_*=2.15(5), *D*=+7.7(1) cm^−1^ and *E*/*D*=+0.026 (Figure S5) in reasonable agreement with the magnetic susceptibility results. According to high‐field EPR spectroscopy, low‐spin manganese(III) complexes with N_4_O_2_ donor or two N_3_ scorpionato ligands exhibit a larger zero‐field splitting with *D*=+19.6/+17.97/ +15.89 cm^−1^ and *E*/*D*=+0.103/+0.023/+0.003.19h, 46, 47

With *χT* of the low‐ and high‐spin complexes set to the spin‐only values 1.00 and 3.00 cm^3^ K mol^−1^, respectively, the experimental magnetic data can be fit to a Boltzmann distribution between the low‐spin and high‐spin complexes without taking into account any cooperativity^48^ with Δ*H*=10.49(4) kJ mol^−1^ and Δ*S*=36.4(1) J mol^−1^ K^−1^ (Supporting Information, Figure S4 b). The critical temperature is close to room temperature (*T*_1/2_=288 K, 50 % high‐spin). The SCO is incomplete up to 350 K (ca. 68 % high‐spin). As the entropy change resulting from the different spin multiplicity amounts to only Δ*S*(spin)=R ln((2*S*_high‐spin_+1)/(2*S*_low‐spin_+1))=R ln(5/3)=4.25 J mol^−1^ K^−1^, the remaining entropic part of ca. 30 J mol^−1^ K^−1^ (88 %) must be attributed to additional vibrational degrees of freedom in the Jahn–Teller distorted high‐spin state. Interestingly, the entropy change Δ*S*=39.4 J mol^−1^ K^−1^ for *trans*‐CrI_2_(depe)_2_ is very similar to that of [Cr(ddpd)_2_][BF_4_]_2_.^10^

Temperature‐dependent IR spectra of KBr pellets of [Cr(ddpd)_2_][BF_4_]_2_ recorded between 10 and 290 K display non‐linear shifts of the IR absorption bands consistent with the gradual SCO obtained from the magnetic data (Supporting Information, Figures S6–S9, Table S1). Furthermore, the temperature‐dependent IR band shifts are fully reversible (Figure S10).

[Cr(ddpd)_2_]^2+^ is one of the very rare chromium(II) spin crossover compounds reported so far and to the best of our knowledge the only one with nearly octahedral symmetry and *T*_1/2_≫200 K (Scheme 1). Consequently, the reduced molecular ruby [Cr(ddpd)_2_]^2+^ represents a textbook example of SCO in the d^4^ electron configuration.

DFT calculations (CPCM(acetonitrile)‐RIJCOSX‐B3LYP‐D3BJ‐ZORA/def2‐TZVPP) of [Cr(ddpd)_2_]^2+^ confirm similar energies for both low‐ and high‐spin complexes in their respective optimized geometries as required for an SCO situation (Δ*G*_298_(low‐spin–high‐spin)=6 kJ mol^−1^). The high‐spin isomer (^5^E term in octahedral symmetry) exhibits elongated Cr−N bonds due to the population of antibonding orbitals and depopulation of weakly bonding orbitals but, most importantly, strongly differing Cr−N bond lengths due to the Jahn–Teller distortion towards an elongated octahedron (Figure 1 b).^40^, ^41^, ^42^ In addition to the simple Cr−N distance elongation by 0.227 Å, the two terminal pyridine rings of one ddpd ligand tilt with Cr‐N‐C_para_ angles of 144°, further reducing the Cr−N interaction (Figure 1 b). The calculated distortion of the high‐spin complex is larger than the distortion observed at 263 K by XRD (Figure 1 b; Supporting Information, Figure S11, Table S2). This is consistent with the estimation that only a fraction of the complexes (ca. 40 %) has undergone SCO to the high‐spin configuration at this temperature according to the magnetic data.

In the low‐spin case, all DFT calculated Cr−N distances are shorter and very similar (Figure 1 b). The Mulliken spin densities at chromium of 2.28 and 3.94 for the low‐ and high‐spin complexes, respectively, further substantiate the metal‐centered SCO without significantly shifting electron/spin density to the ddpd ligand(s) (Supporting Information, Figure S12).

A temperature‐dependent single‐crystal diffraction study fully confirms the geometric changes during the SCO with a higher symmetry at low temperature (120 K) and a beginning Jahn–Teller elongation at higher temperature (263 K) (Figure 1). The metrics determined at 120 K are fully consistent with a low‐spin chromium(II) ion and incompatible with a ligand radical coordinated to Cr^III^ (Figure 1 a; Supporting Information, Table S2).^33^, ^34^

The SCO transition of [Cr(ddpd)_2_][BF_4_]_2_ is accompanied by a reversible color change from pale‐green (>60 % high‐spin) at 343 K to deep green (low‐spin) at 203 K) in solution (Figure 2). The UV/Vis/NIR spectra recorded at different temperatures in ^*n*^PrCN display isosbestic points confirming the clean transformation between the spin isomers. Time‐dependent DFT calculations of the high‐ and low‐spin complexes reproduce the spectra at high and low temperature, respectively (Supporting Information, Figures S13 and S14). The characteristic absorption band pattern of the low‐spin state between 600–900 nm comprises three allowed metal‐to‐ligand charge transfer transitions according to the calculations (calcd 678, 755, 831 nm, Figure S13 b). This agreement between experimental and TD‐DFT derived electronic transitions substantiates the description of the electronic nature of [Cr(ddpd)_2_]^2+^ as a low‐spin chromium(II) ion (^3^T_1_) at low temperature.

**Figure 2:**
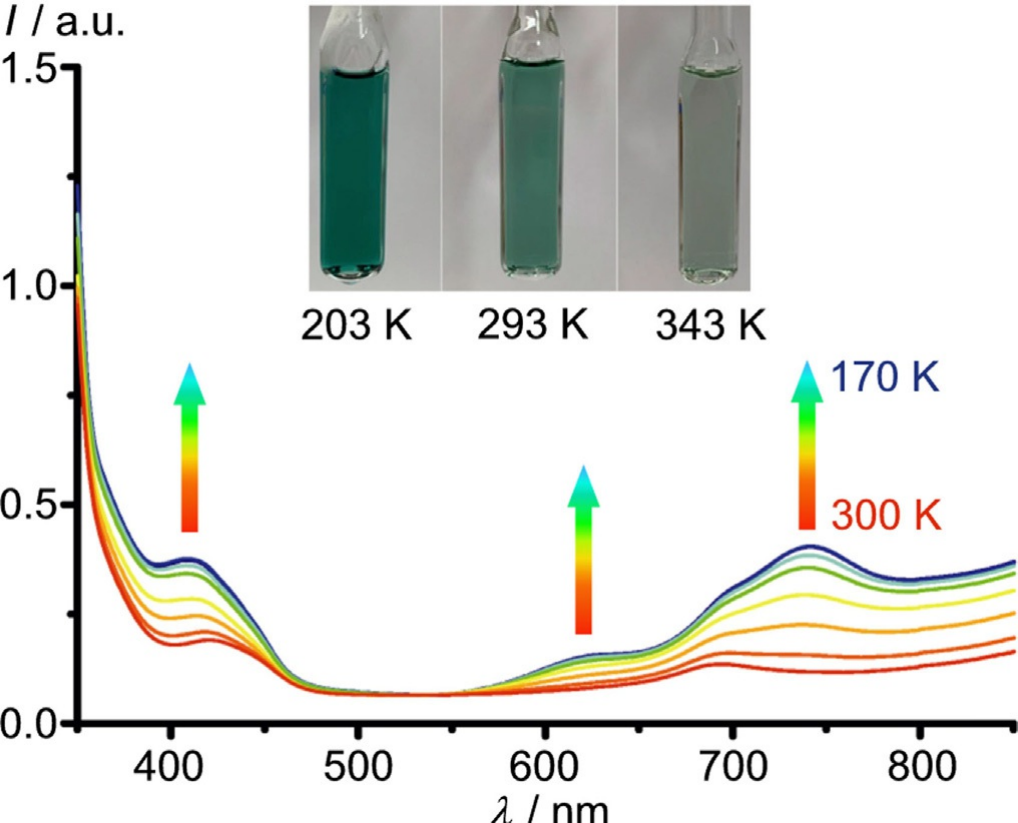
Temperature‐dependent UV/Vis/NIR spectra of [Cr(ddpd)_2_][BF_4_]_2_ in ^*n*^PrCN and photographs of the CH_3_CN solution at 203, 293 and 343 K.

The thermodynamic quantities in ^*n*^PrCN as obtained from fitting the low‐spin and high‐spin fractions are estimated as Δ*H*=24.2(4) kJ mol^−1^ and Δ*S*=95(1) J mol^−1^ K^−1^ (Supporting Information, Figure S15). Differences of the thermodynamic SCO data in solution and the solid state have been noted before for iron(II) and cobalt(II) SCO complexes.^49^, ^50^

The ligand‐centered photoredox chemistry of [Cr(bpy)_3_]^3+^, [Cr(phen)_3_]^3+^, [Cr(tpy)_2_]^3+^ and [Cr(tpe)_2_]^3+^ contrasts the metal‐centered redox and photoredox chemistry of [Cr‐ (ddpd)_2_]^3+^.^33^, ^34^, ^35^, ^36^, ^37^, ^38^, ^39^ This implies different electronic couplings of the various chromium(III) complexes and a reducing agent and consequently different kinetic barriers to the electron transfer. Furthermore, electron transfer to the chromium center in [Cr(ddpd)_2_]^3+^ is affected by the resulting magnetic state of the chromium(II) ion. The thermal SCO of the chromium(II) complex [Cr(ddpd)_2_]^2+^ (^3^T_1_/^5^E) and the ground and lowest excited states of [Cr(ddpd)_2_]^3+^ (^4^A_2_ ground state; ^2^T_1_ excited state) can be combined in the square scheme shown in Figure 3. SCO of the Cr^II^ complexes connects the low‐spin and high‐spin isomers. Excitation with light and luminescence links the ^4^A_2_ and ^2^T_1_ states of the Cr^III^ complex.^23^, ^28^ Finally, single‐electron transfer processes complete the square scheme from these two spin‐inversion reactions. The reduction potential of [Cr(ddpd)_2_]^3+^ in its ^4^A_2_ ground state and the ^2^T_1_ excited state energy are experimentally accessible.^23^, ^28^ Because the Gibbs free energy change of the SCO reaction is close to zero at room temperature, the equilibrium constant is close to unity, and equal populations of both spin states are expected. However, the required large reorganization of the high‐spin state (^5^E) along the Jahn–Teller axis will kinetically hamper the direct formation of this state from the excited ^2^T_1_ state of chromium(III). Reduction of the ^2^T_1_ state to the low‐spin chromium(II) complex (^3^T_1_), however, is not associated with large reorganization barriers as the Cr−N distances remain essentially constant.^23^, ^40^ These thermodynamic and kinetic considerations on redox and photoredox chemistry of [Cr(ddpd)_2_]^3+^ explain its reluctant ground and excited state redox reactivity (in the high‐spin state) and the favored energy transfer reactions in its electronically excited states.^25^

**Figure 3:**
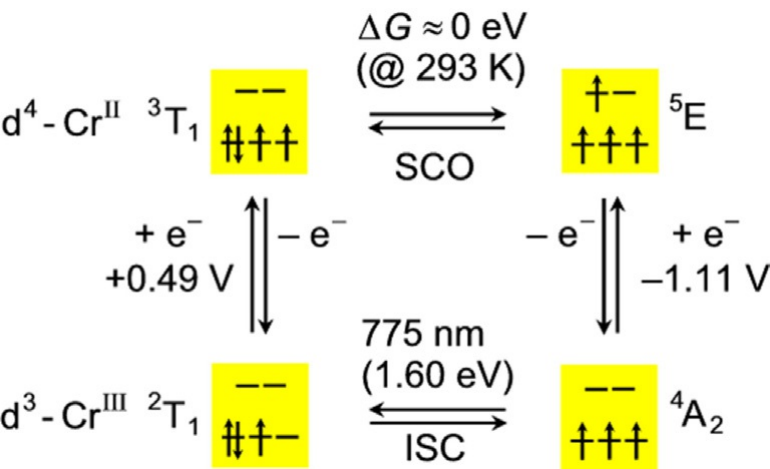
Square scheme of chromium(II) and chromium(III) complexes in their respective low‐ and high‐spin configurations.

Finally, to probe conceivable long‐lived excited states of [Cr(ddpd)_2_]^2+^ at low and high temperature, step‐scan FT‐IR^39^, ^41^, ^52^ spectra of KBr pellets of [Cr(ddpd)_2_][BF_4_]_2_ were recorded at 290 K (ca. 50 % low‐spin) and at 20 K (>98 % low‐spin) between 0–750 ns after excitation with a 355 nm pulse (Figure 4, Supporting Information, Figure S16). Step‐scan FT‐IR spectra were indeed observed for the chromium(II) complexes for the first time, suggestive of long‐lived excited states. From the time‐resolved IR data at low and high temperature, biexponential decays are extracted as 213±12 μs (97 %)/2.0±0.1 μs (3 %) at 20 K and as 8.7±0.3 μs (82 %)/0.52±0.02 μs (18 %) at 290 K (Figure S17). As an unknown amount of the excited species might relax to the ground states at time scales below 50 ns (instrumental time resolution), especially at room temperature, quantum yields cannot be given. However, the long lifetimes of the spectroscopically observed excited states suggest a different multiplicity with respect to the initial states.

**Figure 4:**
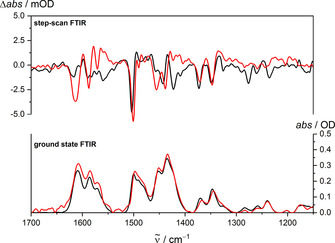
Step‐scan FT‐IR spectra of [Cr(ddpd)_2_][BF_4_]_2_ from 0–750 ns after excitation at *λ*_exc_=355 nm at 20 K (black) and 290 K (red) (top) and corresponding ground state FT‐IR spectra (bottom). At 20 K, essentially the low‐spin state is populated, while at 290 K low‐ and high‐spin states are populated (Supporting Information, Figures S7–S10).

Charge transfer states or ligand field excited states are conceivable candidates. The exact nature of these long‐lived excited states is not yet fully established and further time‐resolved techniques (pump‐probe UV/Vis, XAS, XES) are required, yet application of these probe methods is beyond this initial study.

If the long‐lived states would have ligand field character with different multiplicity than the ground state (triplet vs. quintet), this photoinduced transformation parallels the famous light‐induced excited spin state trapping (LIESST/reverse LIESST) effect of certain iron(II) spin crossover complexes, yet with comparably short lifetime.^2^, ^3^, ^4^, ^53^ The presumably faster relaxation of chromium(II) than iron(II) SCO systems is very likely associated with the smaller change in multiplicity (Cr^II^: Δ*S*=1; Fe^II^: Δ*S*=2) and the corresponding smaller overall structural reorganization. Additionally, the low‐spin isomer possesses several singlet excited states around 8000–10 000 cm^−1^ (^1^E, ^1^T_2_) as obtained from CASSCF(8,12)/NEVPT2 calculations. These also qualify as long‐lived candidate states. The ligand field excited states of low‐ and high‐spin isomers of [Cr(ddpd)_2_]^2+^ are depicted in Figure 5 (Supporting Information, Tables S3–S6).

**Figure 5:**
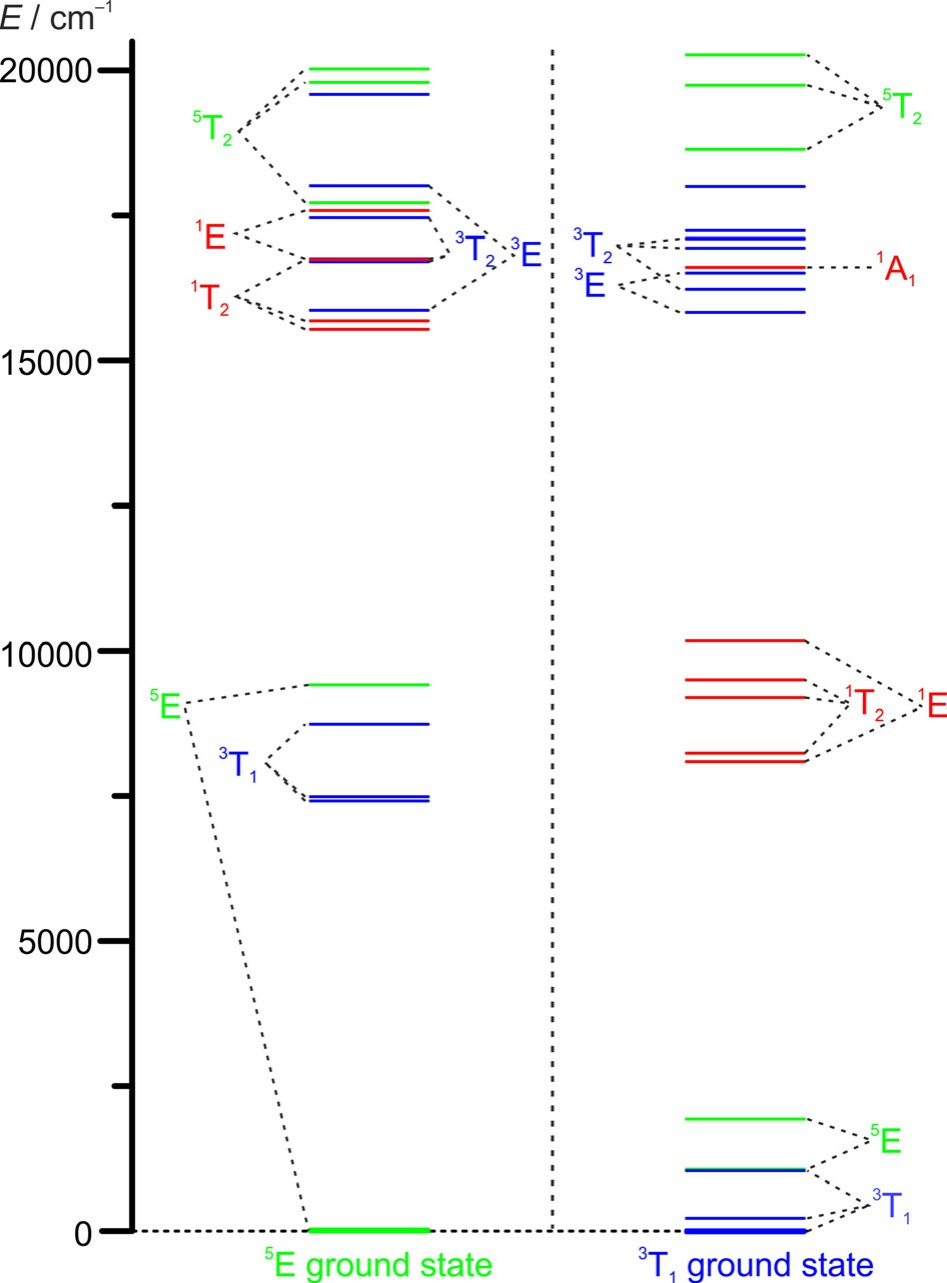
CASSCF(8,12)‐FIC‐NEVPT2 calculated states of [Cr(ddpd)_2_]^2+^ with *S=*2 (^5^E ground state) and *S=*1 (^3^T_1_ ground state) based on DFT optimized geometries of high‐ and low‐spin states, respectively.

In summary, we reported a six‐coordinate polypyridine chromium(II) complex displaying gradual thermal spin crossover in the solid state and in solution. The low‐spin complex is highly symmetric, while the high‐spin complex shows a Jahn–Teller distortion towards an elongated octahedron. UV/Vis/NIR and IR spectroscopic patterns parallel the change of the magnetic susceptibility with temperature. Irradiation of the complexes at 20 and 290 K with 355 nm pulses yields electronically excited states of chromium(II) with μs lifetimes.

Future investigations target the tuning of the transition temperature, the potential cooperativity and completeness (e.g., by modification of counter ions and ligands) and the pressure dependence of the thermal SCO as well as a deeper study of the suggested photoinduced SCO of chromium(II) in addition to expanding the emerging class of SCO complexes based on chromium(II).

## Experimental Section

Experimental synthetic and spectroscopic details can be found in the Supporting Information.

Deposition Number 1958093 ([Cr(ddpd)_2_][BF_4_]_2_ **⋅**2 CH_3_CN) contains the supplementary crystallographic data for this paper. These data are provided free of charge by the joint Cambridge Crystallographic Data Centre and Fachinformationszentrum Karlsruhe Access Structures service www.ccdc.cam.ac.uk/structures.

## Conflict of interest

The authors declare no conflict of interest.

## Supporting information

As a service to our authors and readers, this journal provides supporting information supplied by the authors. Such materials are peer reviewed and may be re‐organized for online delivery, but are not copy‐edited or typeset. Technical support issues arising from supporting information (other than missing files) should be addressed to the authors.

Supplementary
