# Supplementary material for: Spin Crossover and Long‐Lived Excited States in a Reduced Molecular Ruby
Source: Chemistry. 2020 May 20;26(32):7199–204. doi: 10.1002/chem.202001237 (PMC7318154; doi:10.1002/chem.202001237)
Supplement: Supplementary file 1 — Supplementary [file CHEM-26-7199-s001.pdf]

Chemistry–A European Journal

Supporting Information

Spin Crossover and Long-Lived Excited States in a Reduced Molecular Ruby

Patrick M. Becker,[a] Christoph Förster,[a] Luca M. Carrella,[a] Pit Boden,[b] David Hunger,[c] Joris van Slageren,[c] Markus Gerhards,[b] Eva Rentschler,[a] and Katja Heinze*[a]

General Procedures. Diethyl ether was distilled under argon over sodium, acetonitrile over calcium hydride. Butyronitrile was purified according to a published procedure.[S1] The ligand ddpd and $[Cr(NCCH_3)_4][BF_4]_2$ were prepared according to reported procedures.[S2,S3] A glovebox (UniLab/MBraun, Ar 4.8, O_2 < 10 ppm, H_2O < 1 ppm) was used for storage and weighing of sensitive compounds. Reagents were received from usual suppliers (ABCR, Acros Organics, Alfa Aesar, Fischer Scientific, Fluka and Sigma Aldrich). IR spectra were recorded with a Bruker Alpha FT-IR spectrometer with ATR unit containing a diamond crystal. DC magnetic studies were performed with a Quantum Design MPMS-XL-7 SQUID magnetometer on powdered microcrystalline samples. Experimental susceptibility data were corrected for the underlying diamagnetism using Pascal's constants. The temperature dependent magnetic contribution of the holder and of the embedding eicosane matrix was experimentally determined and subtracted from the measured susceptibility data. Variable temperature susceptibility data were collected in a temperature range of 6 – 300 K under an applied field of 0.1 Tesla. UV/Vis/NIR spectra were recorded on a Varian Cary 5000 spectrometer using 1.00 cm cells. High-frequency electron paramagnetic resonance (HFEPR) spectra of pressed powder pellets of $[Cr(ddpd)_2][BF_4]_2$ (7 mg) sealed with Teflon were recorded by means of a homebuilt spectrometer that has been described in the literature.[S4] Simulations were carried by using the Easyspin tool.[S5] The resonances of the $[Cr(ddpd)_2]^{3+}$ impurity were modelled as reported in the literature.[S4b] All time-resolved FT-IR experiments were performed with an FT-IR spectrometer Bruker Vertex 80v, operated in the step-scan mode. KBr pellets of $[Cr(ddpd)_2][BF_4]_2$ (ca. 0.75 mg) were prepared by mixing with dry KBr (ca. 200 mg, stored at 80 °C) and grinding to a homogeneous mixture. The strongest peak in the ground state spectrum showed an absorption of about 0.6 OD with the mentioned concentration. Measurements with cryogenically cooled KBr pellets (20 K and 290 K at the sample) were performed with a closed cycle helium cryostat (ARS Model DE-202A). The cryo cooler was equipped with a homebuilt pellet holder and CaF_2 windows. A liquid-nitrogen-cooled mercury cadmium telluride (MCT) detector (Kolmar Tech., Model KV100-1-B-7/190) with a rise time of 25 ns, connected to a fast preamplifier and a 14-bit transient recorder board (Spectrum Germany, M3I4142, 400 MS s^{-1}), was used for signal detection and processing. The laser setup includes a Q-switched Nd:YAG laser (Innolas SpitLight Evo I) generating pulses with a band-width of 6 — 9 ns at a repetition rate of 100 Hz. The third harmonic (355 nm) of the Nd:YAG laser was used for sample excitation. The UV pump beam was attenuated to about 2.0 mJ per shot at a diameter of 9 mm. The beam was directed onto the sample and adjusted to have a maximal overlap with the IR beam of the spectrometer. The sample chamber was equipped with anti-reflection-coated germanium filters to prevent the entrance of laser radiation into the detector and interferometer compartments. The time delay between the start of the experiment and the UV laser pulse was controlled with a Stanford Research Systems DG535 delay generator. A total number of 5000 (20 K) and 5200 (290 K) coadditions were recorded at each interferogram point. The time resolution was set to 50 ns (20 K) or 10 ns (290 K) and the spectral region was limited by undersampling to 988 – 1975 cm^{-1} with a spectral resolution of 4 cm^{-1} resulting in 555 interferogram points. An IR broad band filter (850 – 1750 cm^{-1}) and the CaF_2 windows (no IR transmission < 1000 cm^{-1}) of the cryostat prevented problems when performing a Fourier transformation (*i.e.* no IR intensity outside the measured region should be observed). FT-IR ground state spectra were recorded systematically to check for sample degradation. A more detailed description of the step-scan setup is given here.[S6-S8]

Crystal structure determinations. Diffusion of diethyl ether into concentrated solutions of $[Cr(ddpd)_2][BF_4]_2$ in CH_3CN yielded diffraction quality crystals. Intensity data were collected with a STOE IPDS-2T diffractometer with an Oxford cooling using Mo K_α radiation (λ = 0.71073 Å). The diffraction frames were integrated using the STOE X-Area software package[S9] and most were corrected for

absorption with MULABS[S10] of the PLATON software package[S11]. The structures were solved by direct methods and refined by the full-matrix method based on F^2 using the SHELXL software package[S12] using the ShelXle graphical interface[S13]. All non-hydrogen atoms were refined anisotropically, while the positions of all hydrogen atoms were generated with appropriate geometric constraints and allowed to ride on their respective parent atoms with fixed isotropic thermal parameters.

CCDC 1958093 ($[Cr(ddpd)_2][BF_4]_2\times2CH_3CN$) contains the supplementary crystallographic data for this paper. These data are provided free of charge by The Cambridge Crystallographic Data Centre.

Crystallographic Data of *mer*-$[Cr(ddpd)_2][BF_4]_2\times2CH_3CN$: $C_{38}H_{40}B_2F_8CrN_{12}$ (890.44); T = 120 K; orthorhombic; *F*ddd; a = 14.066(3) Å, b = 21.221(4) Å, c = 25.985(5) Å, V = 7756(3) Å^3; Z = 8; density, calcd. = 1.525 g cm^{-3}, μ = 0.382 mm^{-1}; $F(000)$ = 3664; crystal size 0.480 × 0.380 × 0.300 mm; θ = 2.924 to 28.218 deg.; $-17\leq h\leq18$, $-25\leq k\leq28$, $-34\leq l\leq39$; rfln collected = 10114; rfln unique = 2392 [R(int) = 0.0302]; completeness to θ = 25.242 deg. = 99.8 %; semi empirical absorption correction from equivalents; max. and min. transmission 1.148 and 0.884; data 2392; restraints 73, parameters 224; goodness-of-fit on F^2 = 1.162; final indices [$I > 2\sigma(I)$] R_1 = 0.0429, wR_2 = 0.1241; R indices (all data) R_1 = 0.0469, wR_2 = 0.1266; largest diff. peak and hole 0.358 and –0.585 e Å^{-3}.

Density functional theoretical calculations on the chromium complex cations $[Cr(ddpd)_2]^{2+}$ were carried out using the ORCA program package (version 4.0.1).[S14] Tight convergence criteria were chosen for all calculations (keywords tightscf and tightopt). All calculations make use of the resolution of identity (Split-RI-J) approach for the Coulomb term in combination with the chain-of-spheres approximation for the exchange term (COSX).[S15,S16] Geometry optimization was performed using the B3LYP functional[S17] in combination with Ahlrichs' split-valence triple-ζ basis set def2-TZVPP for all atoms.[S18,S19] The optimized geometries were confirmed to be local minima on the respective potential energy surface by subsequent numerical frequency analysis (N_{imag} = 0). TD-DFT calculations were performed at the same level of theory. Fifty vertical spin-allowed transitions were calculated. The zero order relativistic approximation was used to describe relativistic effects in all calculations (keyword ZORA).[S20,S21] Grimme's empirical dispersion correction D3(BJ) was employed (keyword D3BJ).[S22,S23] To account for solvent effects, a conductor-like screening model (keyword CPCM) modeling acetonitrile was used in all calculations.[S24] Explicit counterions and/or solvent molecules were neglected.

Complete-active-space self-consistent field calculations in conjunction with N-electron valence perturbation theory to second order (NEVPT2)[S25,S26] in order to recover missing dynamic electron correlation were performed on DFT optimized low- and high-spin geometries of $[Cr(ddpd)_2]^{2+}$. In order to accurately model the ligandfield, active spaces were chosen to encompass the dominant σ-bonding (e_g in O_h symmetry) and ligand-field (t_{2g} and e_g*) orbitals formed between chromium and the ligand. A second d shell[27] was employed in these calculations. An active space of (8,12) along with 5 quintet roots, 11 triplet roots and 11 singlet roots was selected. Without FIC-NEVPT2 correction for electron correlation, the electronic ground state of $[Cr(ddpd)_2]^{2+}$ in the DFT optimized low-spin geometry was incorrectly described as a quintet state. This effect was even more pronounced for small active spaces such as (4,5) (t_{2g}, e_g*) and (8,7) (e_g, t_{2g}, e_g*) lacking the second d shell. Inclusion of metal ligand π-antibonding orbitals (t_{2g}*) or ligand centered π* orbitals failed to better describe the electronic ground state or these orbitals were exchanged by 4d orbitals as active orbitals during CASSCF calculations. Finally, the CASSCF(8,12)-FIC-NEVPT2 calculations were performed using Ahlrichs' split-valence triple-ζ basis set def2-TZVPP for all atoms, except for Cr, which has been described with a quadruple-ζ basis set def2-QZVPP.[S18,S19]

Synthesis of $[Cr(ddpd)_2][BF_4]_2$: Under oxygen-free conditions, ddpd (200 mg, 0.679 mmol) dissolved in acetonitrile (4 ml) was added dropwise to a pale blue solution of $[Cr(CH_3CN)_4][BF_4]_2$ (135 mg, 0.346 mmol) in acetonitrile (20 ml). The reaction mixture turned dark green. After stirring for four hours, the solution was concentrated to about 7 ml under reduced pressure. Diffusion of diethyl ether into the solution gave dark green crystals. Yield: 215 mg (0.266 mmol, 78 %).

UV/Vis (CH_3CN, 295 K): $\lambda(\varepsilon)$ = 399 (1590, sh), 628 (908, sh), 747 (2080), 885 (1870 M^{-1} cm^{-1}) nm. UV/Vis (^{n}PrCN, 295 K): $\lambda(\varepsilon)$ = 399 (1700, sh), 628 (1020, sh), 740 (2270), 880 (2030 M^{-1} cm^{-1}) nm. UV/Vis (^{n}PrCN, 170 K): λ = 408, 628 (sh), 741 nm. IR (ATR, 295 K): $\tilde{\nu}$ = 3102 (w br, CH), 1594 (s, sh), 1581 (s), 1487 (s), 1447 (w), 1429 (vs), 1361 (m), 1331 (s), 1284 (w), 1258 (vw), 1235 (w), 1169 (vw), 1134 (w), 1045 (vs, BF), 1033 (vs, BF), 944 (m), 864 (w), 810 (w), 777 (s), 750 (s), 660 (vw), 637 (w), 616 (w), 579 (m), 519 (s), 454 (vw), 437 (w), 410 (w) cm^{-1}. Magnetism: χT = 2.06 cm^3 K mol^{-1} (295 K); 1.01 (50 K), 0.75 cm^3 K mol^{-1} (4 K) (solvate-free sample). Fit of low temperature data with g = 2.000(3), D = +5.95(12) cm^{-1}. HF-EPR (pellet): $g_{x,y,z}$ = 2.08, 2.10, 2.15, D = +7.7 cm^{-1} and E/D = +0.026 cm^{-1} (5 K).

a) b)

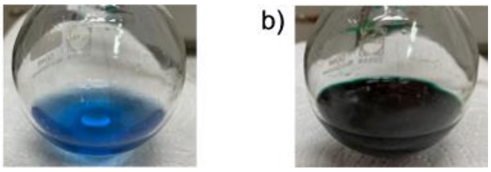

Figure S1. CH_3CN solution of $[Cr(CH_3CN)_4][BF_4]_2$ a) before and b) after addition of ddpd.

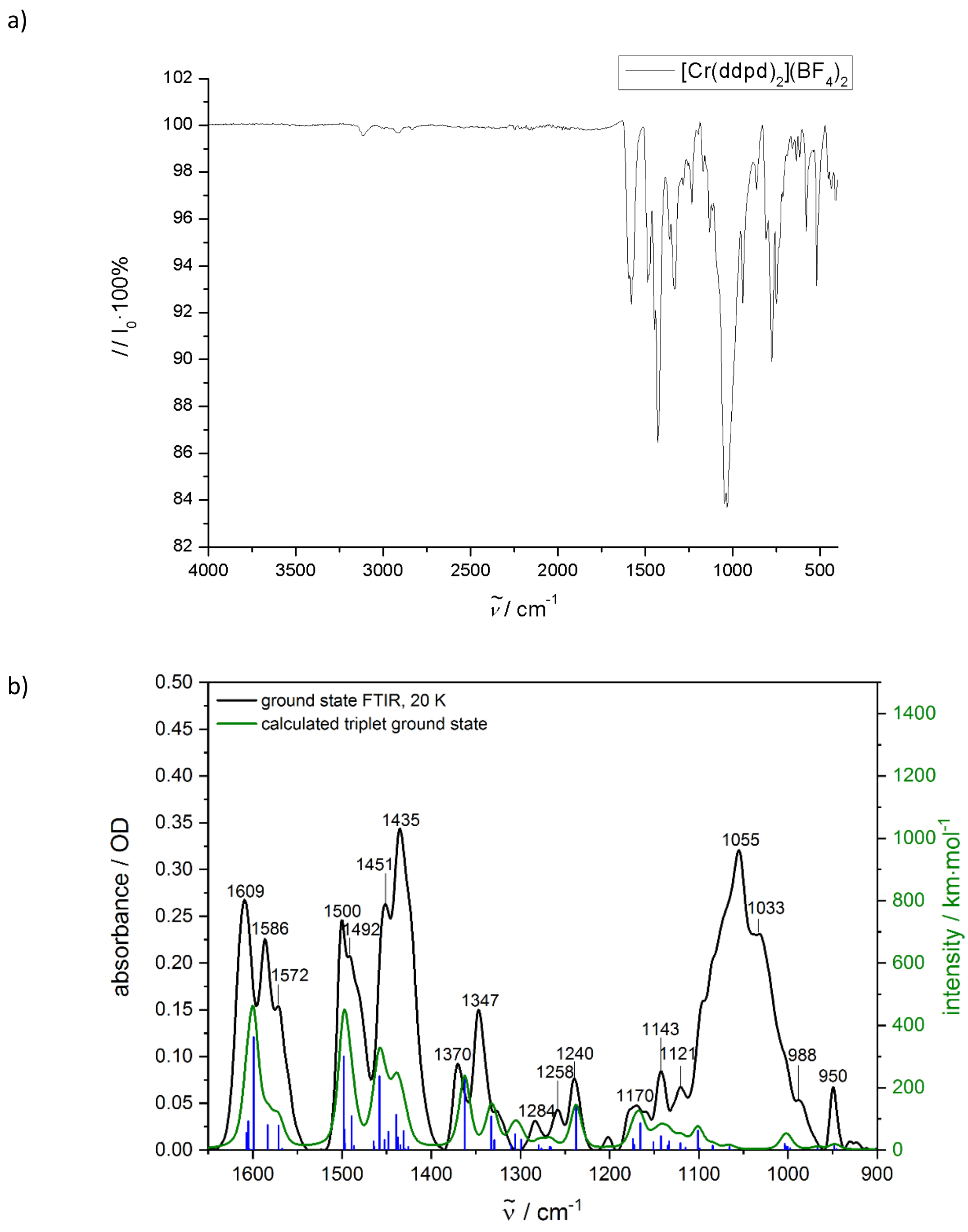

Figure S2. a) ATR-IR spectrum of $[Cr(ddpd)_2][BF_4]_2$ (solvent-free sample) at 295 K and b) ground state FT-IR spectrum of $[Cr(ddpd)_2][BF_4]_2$ (black) (KBr pellet, 20 K), UDFT calculated IR absorption transitions (blue) (scaled by 0.98), and UDFT calculated IR spectrum (green) of $[V(ddpd)_2]^{3+}$ (scaled by 0.98, FWHM = 8 cm^{-1}, gaussian profile

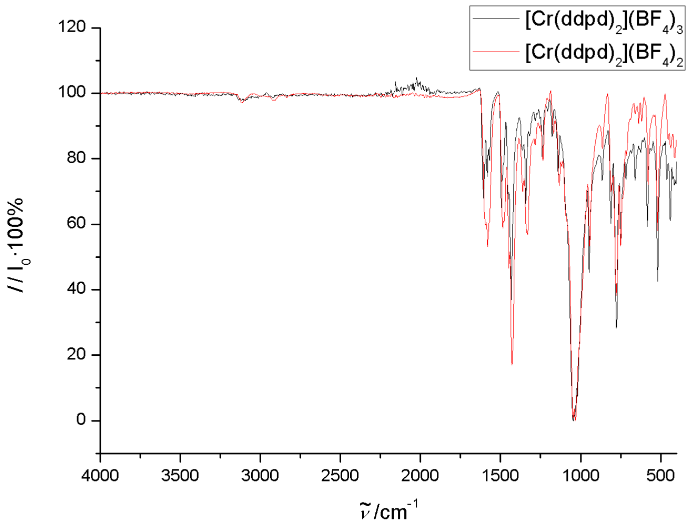

Figure S3. ATR-IR spectra of [Cr(ddpd)$_2$][BF$_4$]$_3$ (black) and [Cr(ddpd)$_2$][BF$_4$]$_2$ (red) normalized to the strong absorption of the BF stretching vibration at 295 K.

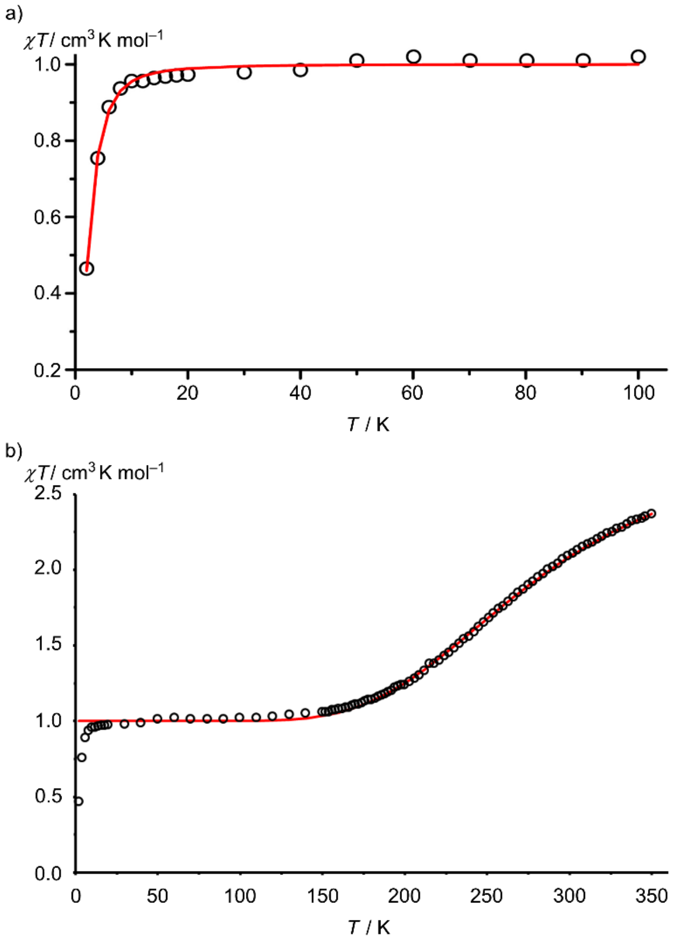

Figure S4. a) χT vs. T plot of [Cr(ddpd)$_2$][BF$_4$]$_2$ (solvent-free sample) from 2 to 100 K; fit with g = 2.000 and D = +5.95 cm^{-1} shown in red. The following spin Hamiltonian was employed: $\mathcal{H} = \mu_B \hat{\mathbf{S}} \cdot \mathbf{g} \cdot \mathbf{B} + D\hat{S}^2$. b) χT vs. T plot of [Cr(ddpd)$_2$][BF$_4$]$_2$ (solvent-free sample) from 2 to 350 K; fit of the data 10 – 350 K to a Boltzmann distribution function with ΔH = 10.49(0.04) kJ mol^{-1} and ΔS = 36.4(0.1) J mol^{-1} K^{-1} (χT(low-spin) = 1.000 cm^3 K mol^{-1} and χT(high-spin) = 3.001 cm^3 K mol^{-1}) shown in red.

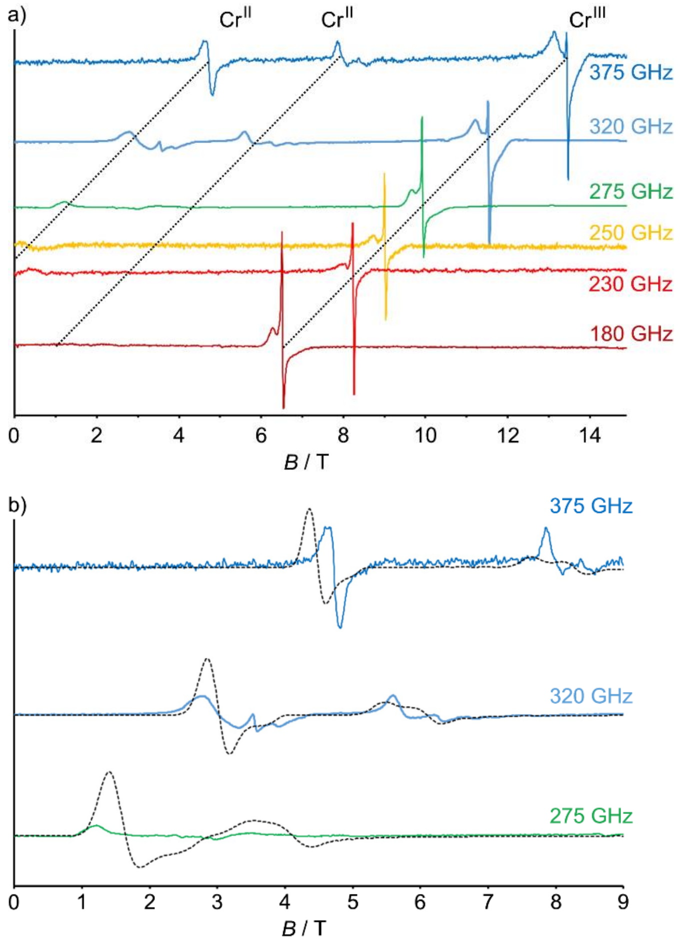

Figure S5. a) HFEPR spectra of a pressed powder pellet of $[Cr(ddpd)_2][BF_4]_2$ (blue) at 5 K and different frequencies as indicated on the vertical axis. The sharp resonance at higher fields corresponds to a $[Cr(ddpd)_2]^{3+}$ impurity.[S4b] b) Simulations of the high frequency spectra of the Cr^{II} region (dotted black) based on the spin Hamiltonian and parameters given below.

$$\mathcal{H} = \mu_B \hat{\mathbf{S}} \cdot \mathbf{g} \cdot \mathbf{B} + D\hat{S}_z^2 + E\left(\hat{S}_x^2 - \hat{S}_y^2\right)$$

S = 1; g_x, g_y, g_z = 2.08, 2.10, 2.15; D = 7.7 cm^{-1}; E = 0.2 cm^{-1}

Table S1. Experimental bands of $[Cr(ddpd)_2][BF_4]_2$ in a KBr pellet and IR transitions (scaled by 0.98) calculated by DFT for $[Cr(ddpd)_2]^{2+}$ (triplet) and assignments.

$\tilde{\nu}_{exp}$ / cm^{-1} at 20 K	$\tilde{\nu}_{calcd.,scaled}$ / cm^{-1} (triplet state)	character of the vibration
1609	1599	aromatic C–C stretching
1586	1583	aromatic C–C and C–N stretching
1572	1571	aromatic C–C and C–N stretching
1500	1498	aromatic C–H bending coupled with aliphatic C–H bending
1492	1490	aromatic C–H bending coupled with aliphatic C–H bending
1451	1458	aromatic C–H bending coupled with aliphatic C–H bending
1435	1440	aromatic C–H bending coupled with aliphatic C–H bending
1370	1362	C–H bending coupled with C–N stretching
1347	1333	C–H bending coupled with C–N stretching
1284	1306	C–H in plane
1258	1299	C–H in plane
1240	1238	C–H in plane
1170	1165	aromatic and aliphatic C–H bending
1143	1143	aromatic and aliphatic C–H bending
1121	1120	aromatic and aliphatic C–H bending
overlap with BF_4^-	1102	aromatic C–H bending
overlap with BF_4^-	1004	pyridine breathing
950	948	C–H bending coupled with C=N and C=C stretching

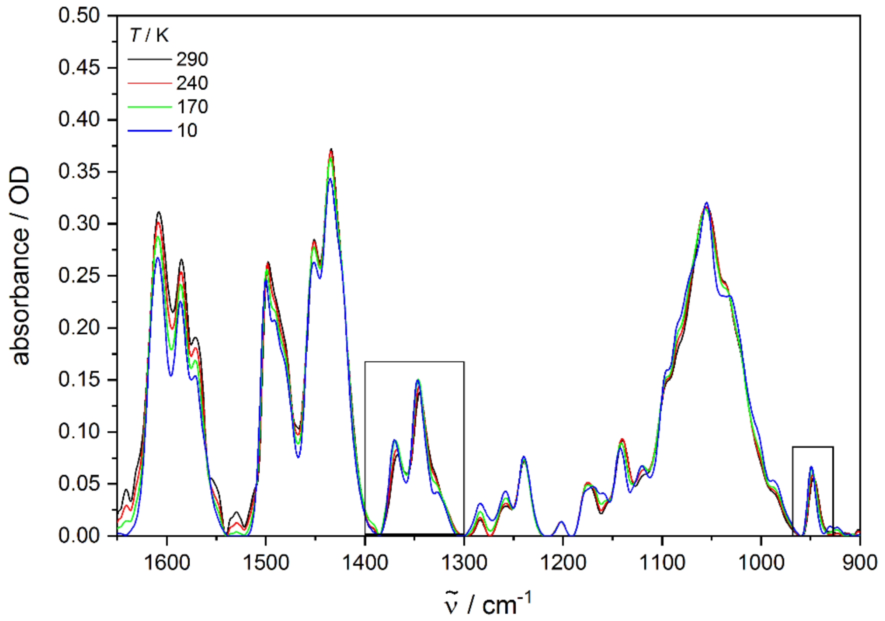

Figure S6. Variable-temperature FT-IR spectra of $[Cr(ddpd)_2][BF_4]_2$ in a KBr pellet. The regions indicated by boxes are displayed in Figures S7 and S8.

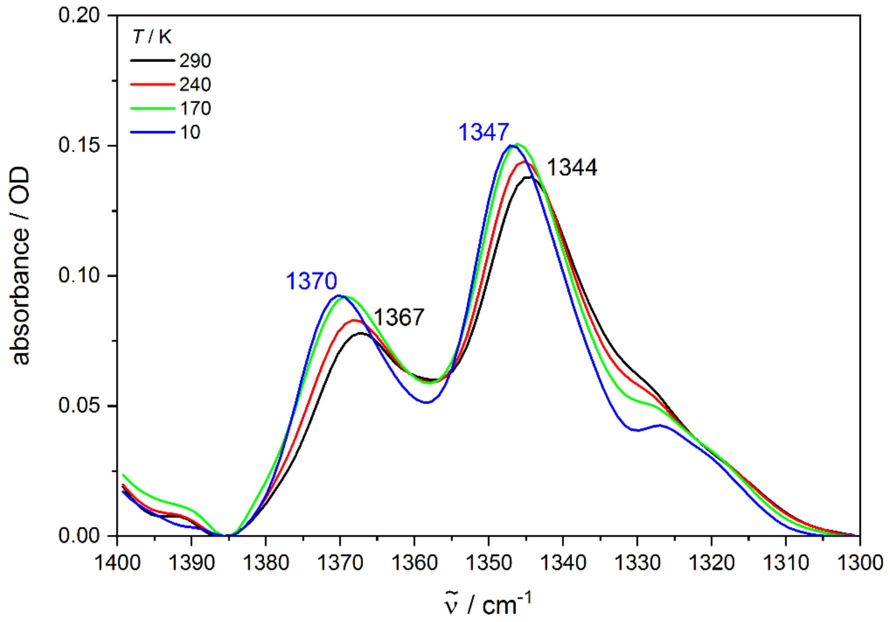

Figure S7. Zoom (1400 – 1300 cm^{-1}) into the variable-temperature FT-IR spectra of $[Cr(ddpd)_2][BF_4]_2$ in a KBr pellet.

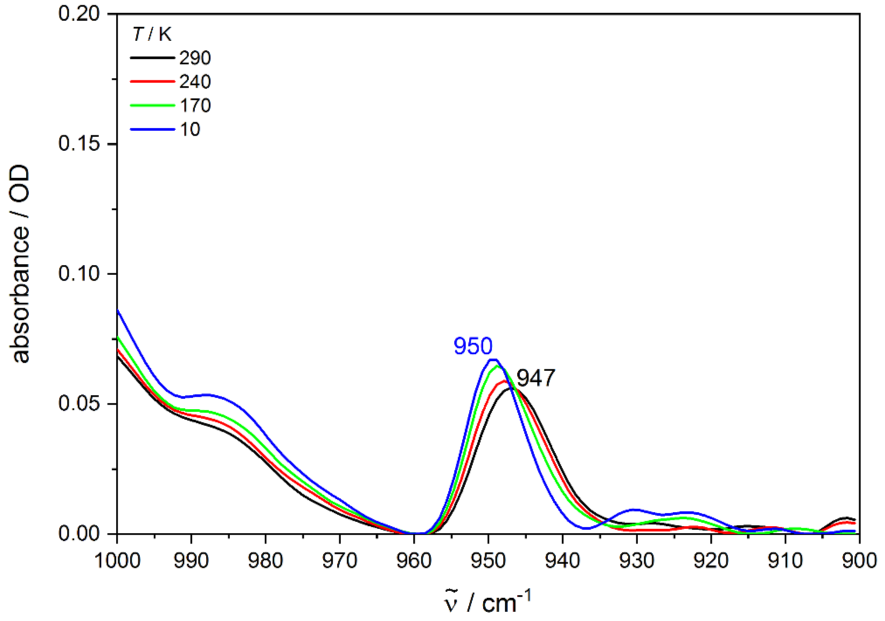

Figure S8. Zoom (1000 – 900 cm^{-1}) into the variable-temperature FT-IR spectra of $[Cr(ddpd)_2][BF_4]_2$ in a KBr pellet.

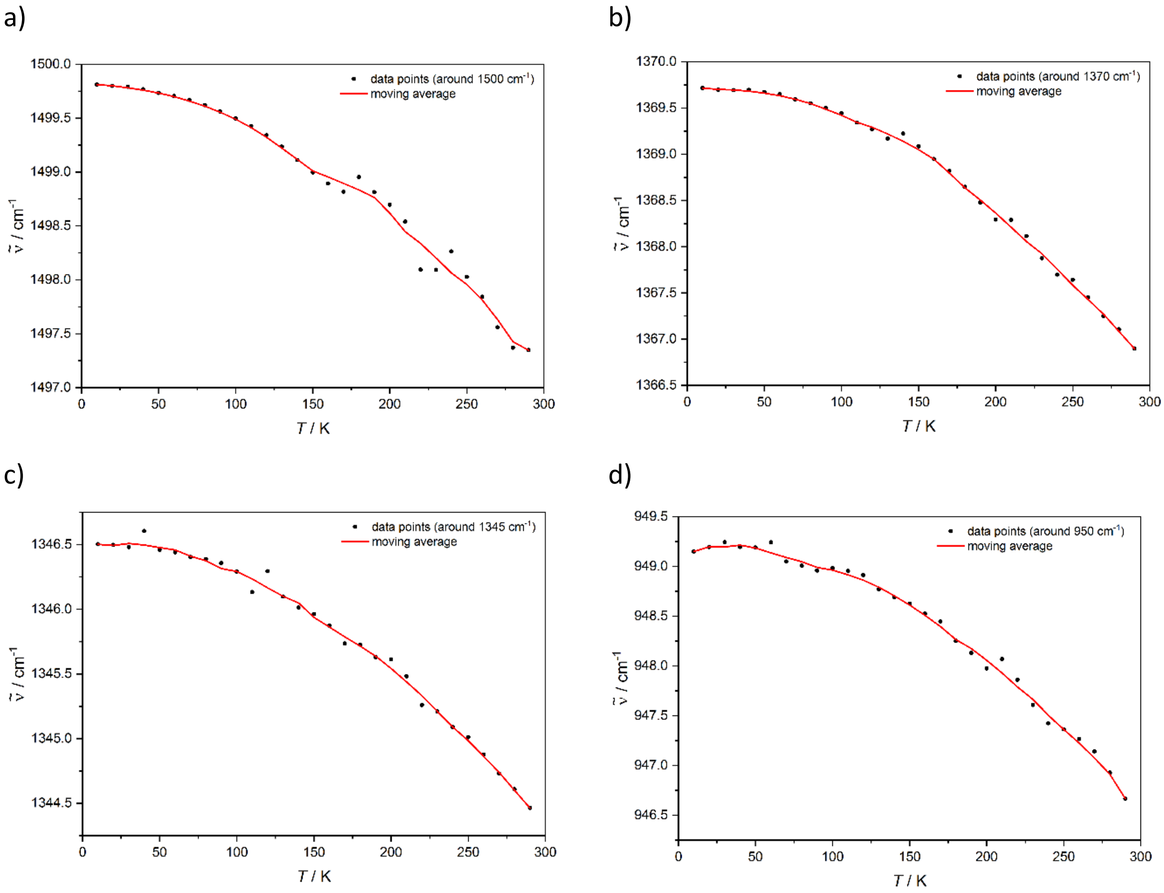

Figure S9. Temperature-dependent shift of selected IR bands at a) ca. 1500 cm^{-1}, b) ca. 1370 cm^{-1}, c) ca. 1345 cm^{-1} and d) ca. 950 cm^{-1}. The red lines are a guide to the eye.

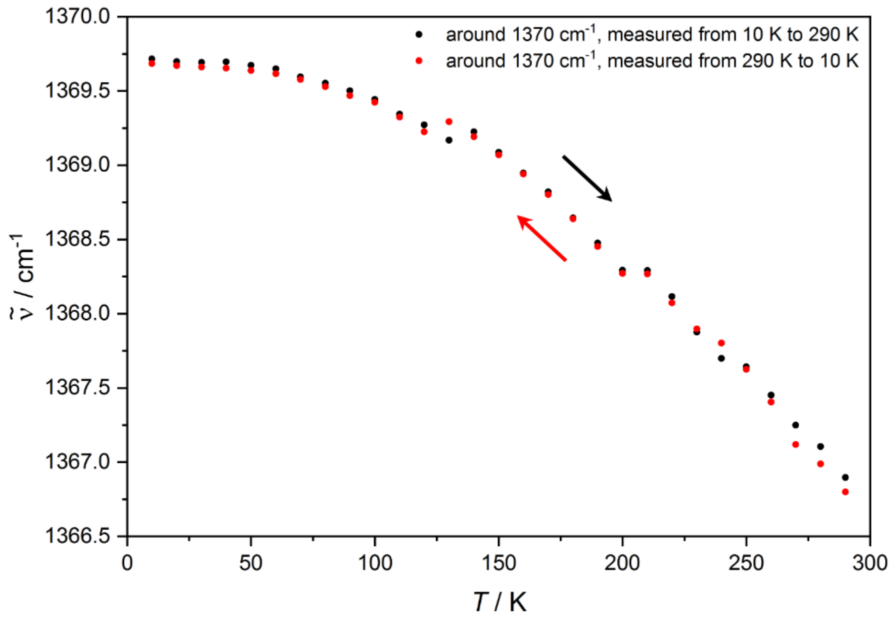

Figure S10. Temperature-dependent shift of the IR band at ca. 1370 cm^{-1} during warming (black) and cooling (red).

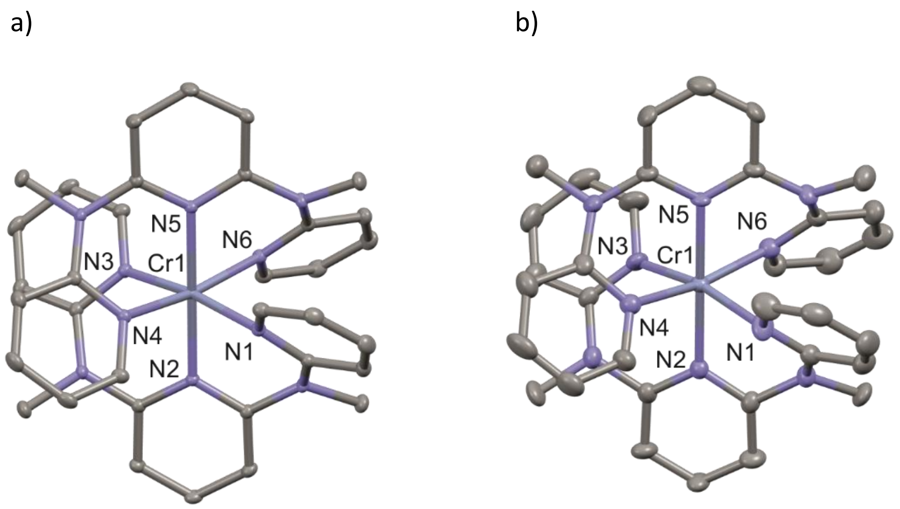

Figure S11. Plots of the cations of $[Cr(ddpd)_2][BF_4]_2 \times 2CH_3CN$ with thermal ellipsoids set at 30 % probability at a) 120 K with N1,N3, N4, N6 and N2, N5 equivalent by symmetry) and b) at 263 K with N1,N3 and N4, N6 equivalent by symmetry.

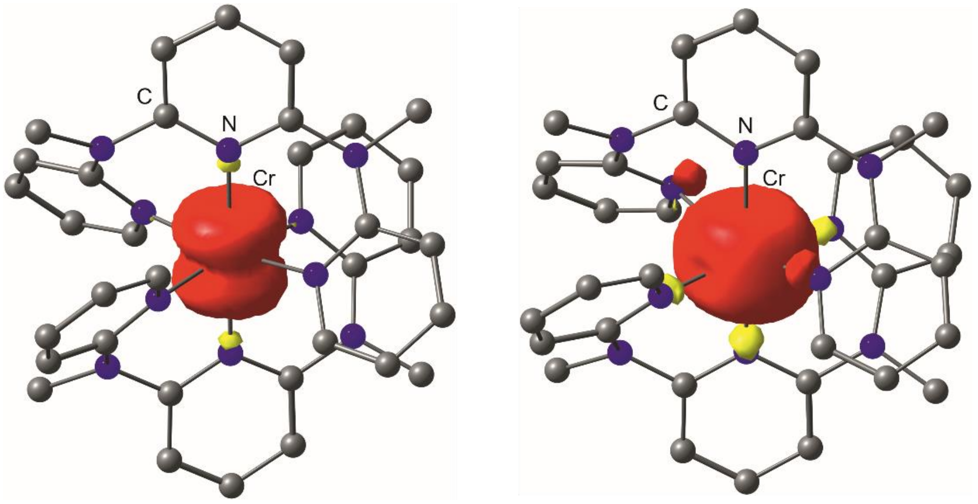

Figure S12. DFT optimized geometries of a) $[Cr(ddpd)_2]^{2+}$ (triplet state; low spin), b) $[Cr(ddpd)_2]^{2+}$ (quintet state; high spin) and corresponding Mulliken spin densities plotted at 0.006 a.u. with the α spin in red and the β spin in yellow.

Table S2. Selected distances (Å) and angles (°) for $[Cr(ddpd)_2][BF_4]_2 \times 2CH_3CN$ at 120 K and 263 K and low-spin and high-spin $[Cr(ddpd)_2]^{2+}$ (DFT). Atom numbering according to Fig. S11.

	XRD		DFT	
	at 120 K	at 263 K	low-spin $[Cr(ddpd)_2]^{2+}$	high-spin $[Cr(ddpd)_2]^{2+}$
Cr1-N1	2.0422(15)	2.117(7)	2.109	2.337
Cr1-N2	2.041(2)	2.070(11)	2.111	2.140
Cr1-N3				
Cr1-N4		2.089(6)		2.107
Cr1-N5		2.064(9)		2.060
Cr1-N6				
ddpd (intraligand)				
N1-Cr1-N2	85.54(4)	83.4(2)	85.3	79.0
N1-Cr1-N3	171.07(9)	166.7(4)	170.7	158.0
N2-Cr1-N3				
N4-Cr1-N5		84.8(2)		85.4
N4-Cr1-N6		169.7(4)		170.7
N5-Cr1-N6				
ddpd (interligand)				
N1-Cr1-N4	89.79(9)	88.73(13)	89.7	87.2
N1-Cr1-N5	94.46(4)	96.6(2)	94.7	101.0
N1-Cr1-N6	90.91(9)	92.47(13)	91.0	94.6
N2-Cr1-N4		95.2(2)		94.7
N2-Cr1-N5	180.0	180.0	180.0	180.0
N2-Cr1-N6				

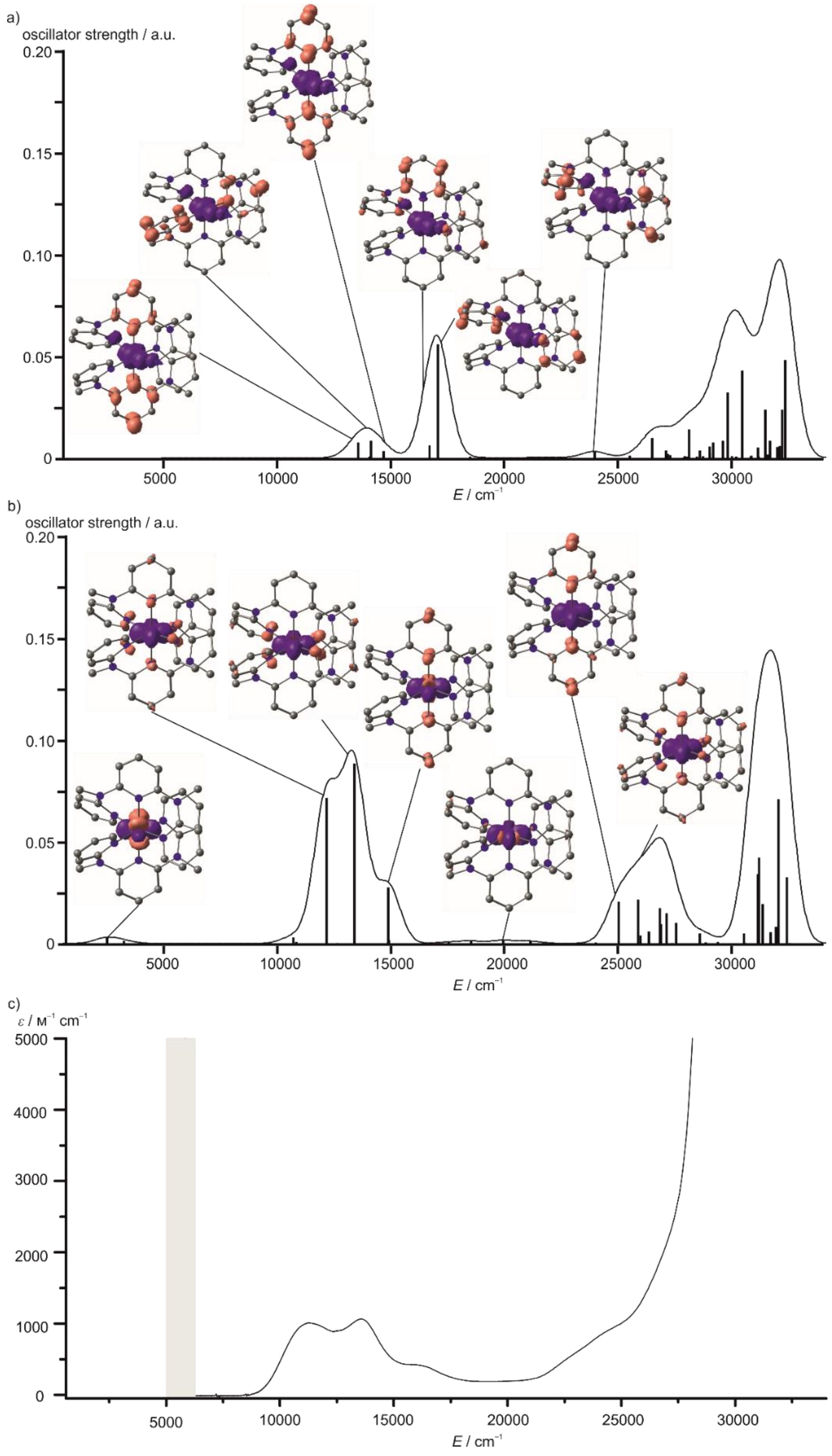

Figure S13. a) TD-DFT calculated 50 vertical transitions and stick spectrum approximated by Gaussian bands with FWHM of 1250 cm^{-1} of geometry optimized high-spin $[Cr(ddpd)_2]^{2+}$ (S = 2) and b) low-spin $[Cr(ddpd)_2]^{2+}$ (S = 1). Difference electron densities of transitions with significant oscillator strength plotted with an isosurface value of 0.005 a.u.; purple = depletion; orange = gain. Hydrogen atoms omitted. c) UV/Vis/NIR spectrum of $[Cr(ddpd)_2][BF_4]_2$ (CH_3CN, T = 295 K). Shaded area omitted due to insufficient baseline correction.

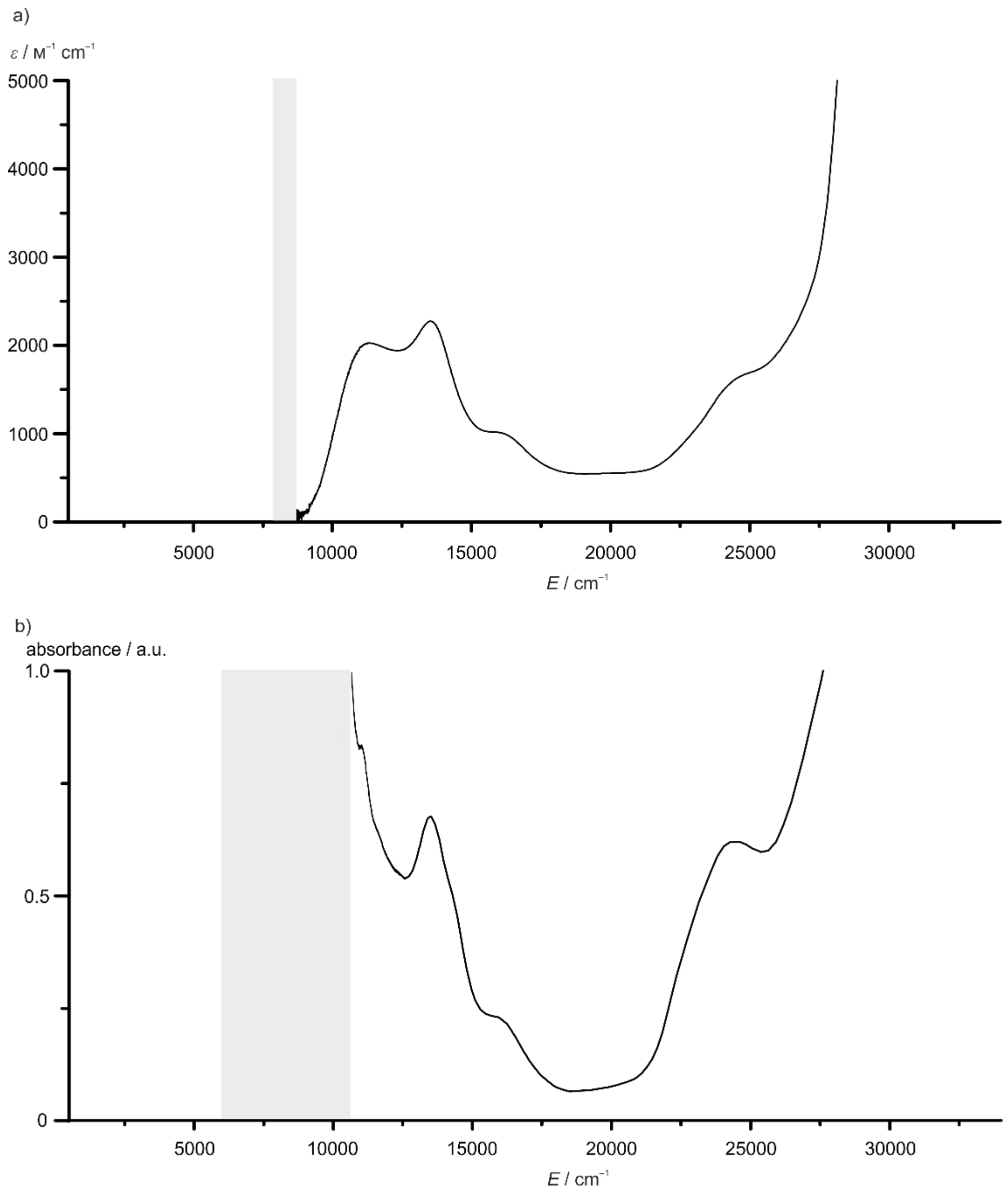

Figure S14. UV/Vis/NIR spectra of $[Cr(ddpd)_2][BF_4]_2$ (^{n}PrCN) at a) T = 295 K and b) T = 170 K (shaded areas omitted due to insufficient baseline correction).

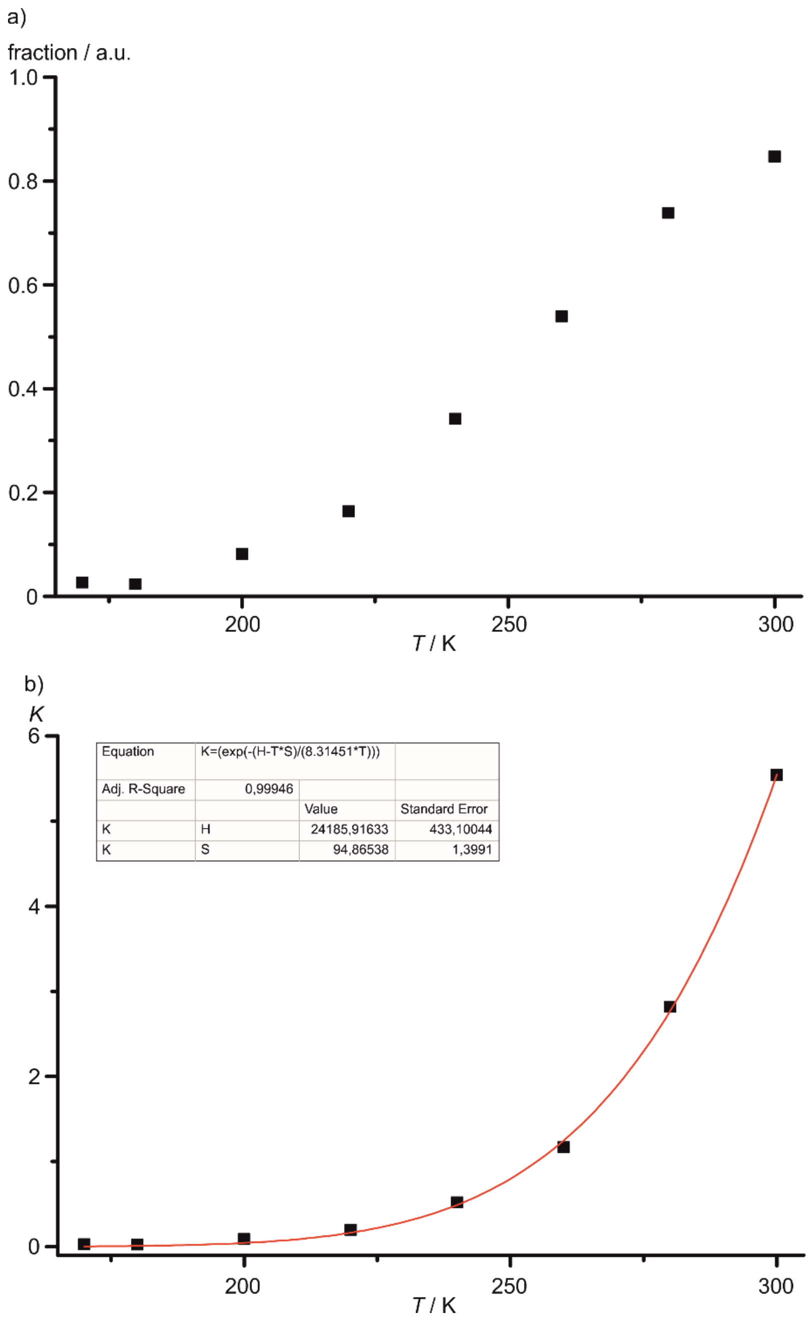

Figure S15. a) High-spin fraction versus temperature and b) fit of the equilibrium constant *K* versus *T* as estimated from the UV/Vis/NIR data at 740 nm in ^{n}PrCN solution with ΔH = 24.2(0.4) kJ mol^{-1} and ΔS = 95(1) J mol^{-1} K^{-1}.

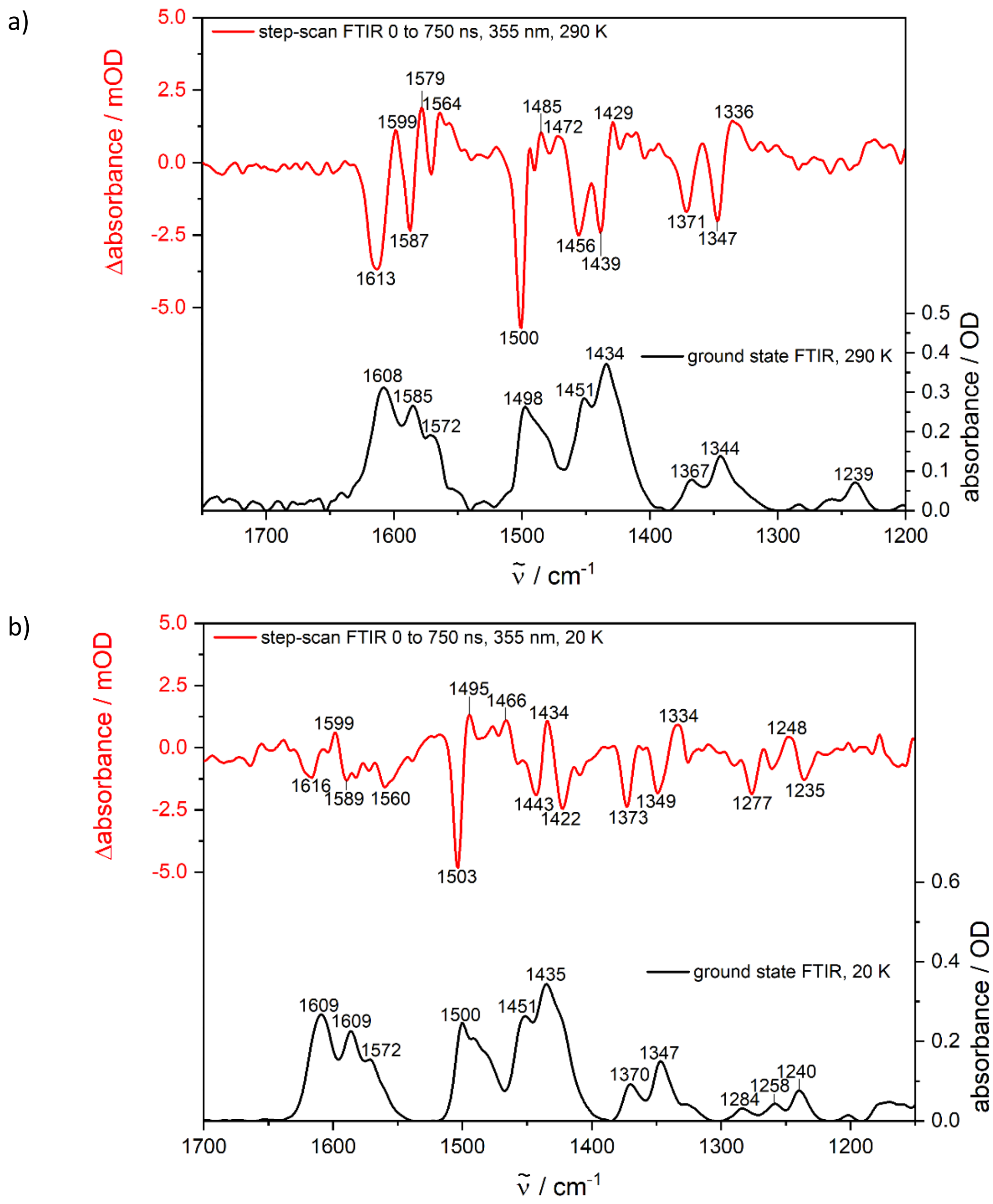

Figure S16. Step-scan FT-IR spectra (red) and ground state FT-IR spectra (black) of $[Cr(ddpd)_2][BF_4]_2$ in a KBr pellet (λ_{exc} = 355 nm; 0 – 750 ns) a) at T = 290 K and b) at T = 20 K.

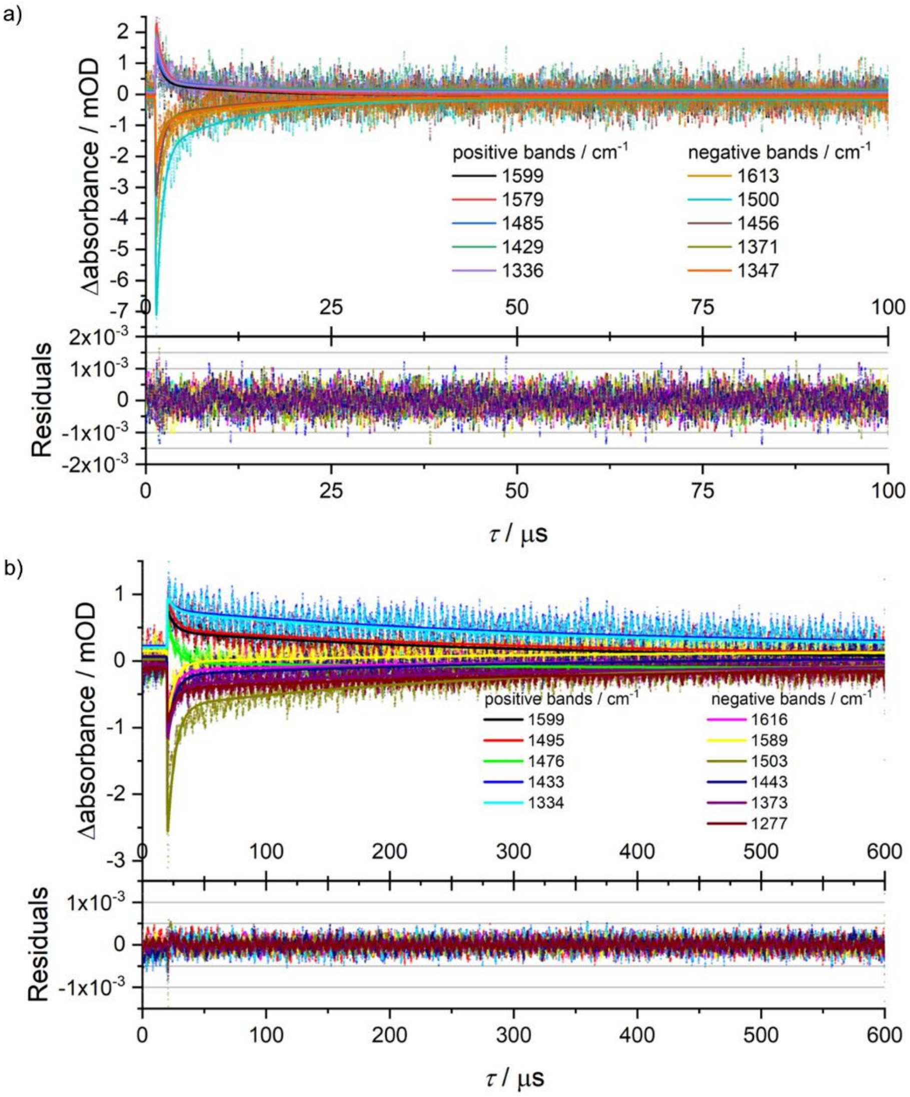

Figure S17. Decay curves, global fits and residuals obtained from step-scan FT-IR data of $[Cr(ddpd)_2][BF_4]_2$ in a KBr pellet a) at 290 K and b) at 20 K.

Table S3. Energies/Hartree and active orbitals used in the CASSCF(8,12)-FIC-NEVPT2 calculations at a contour value of 0.05 a.u. (hydrogen atoms omitted for clarity) of geometry optimized $[Cr(ddpd)_2]^{2+}$ (*S* = 2).

***E* / H**	**orbital**	***E* / H**	**orbital**
–0.5228	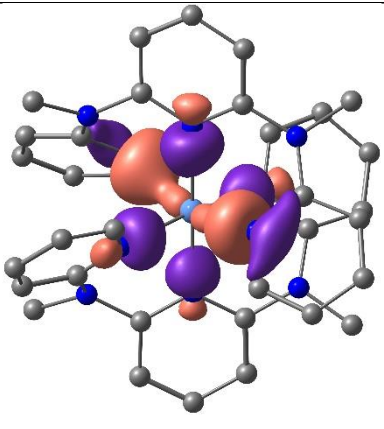	0.2440	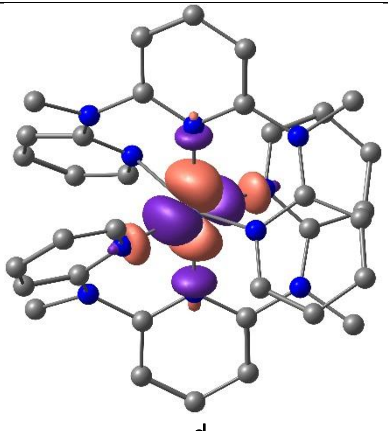 d_{x2-y2}
–0.5410	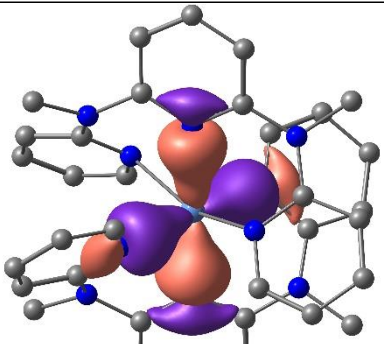	1.1388	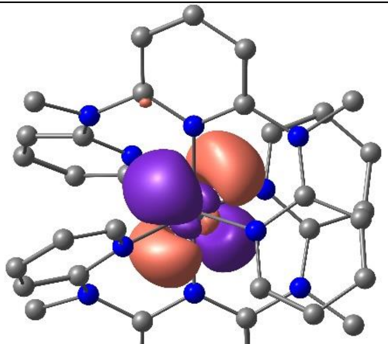
–0.0707	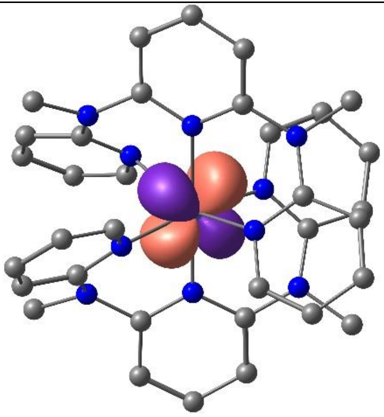 d_{xy}	1.1335	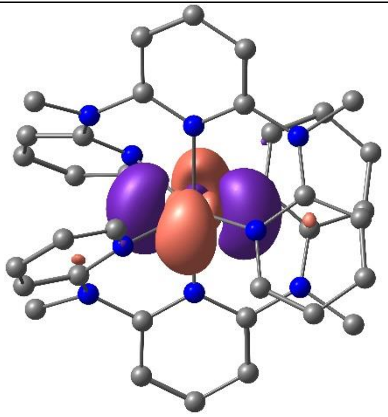

–0.0446	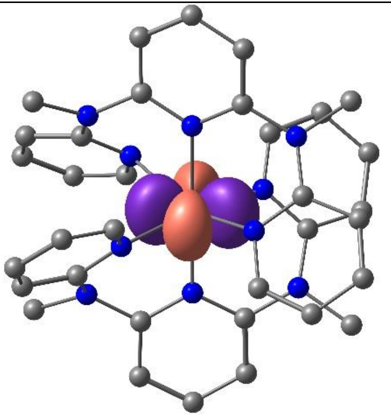 d_{xz}	1.1586	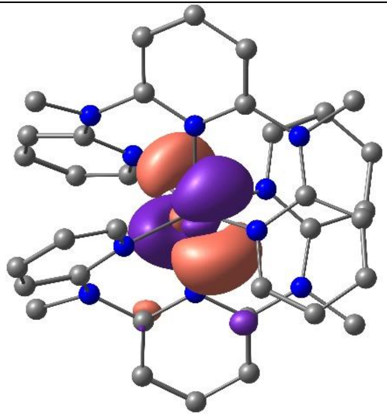
–0.0287	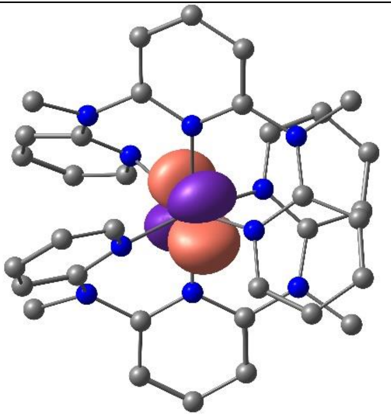 d_{yz}	1.3821	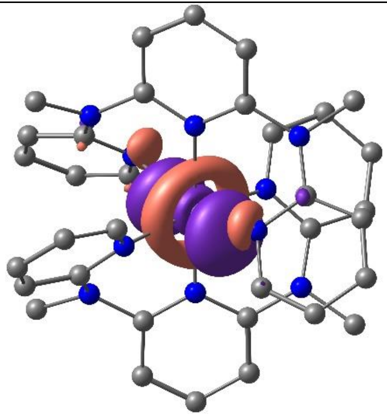
0.1205	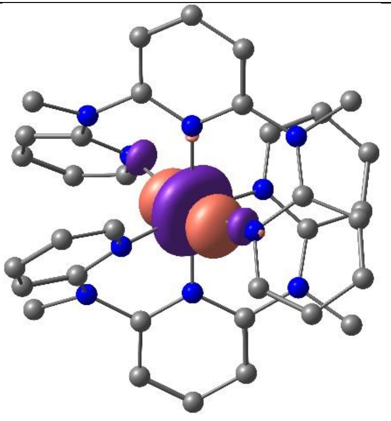 d_{z2}	1.7770	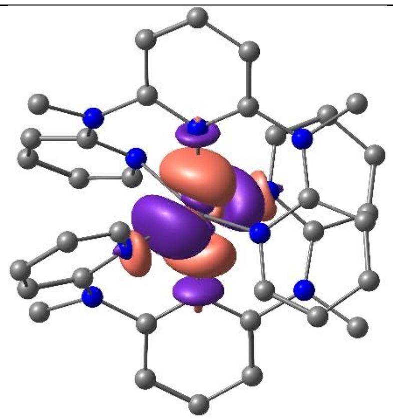

Table S4. Energies/Hartree and active orbitals used in the CASSCF(8,12)-FIC-NEVPT2 calculations at a contour value of 0.05 a.u. (hydrogen atoms omitted for clarity) of geometry optimized $[Cr(ddpd)_2]^{2+}$ (*S* = 1).

E / H	orbital	*E* / H	orbital
–0.5375	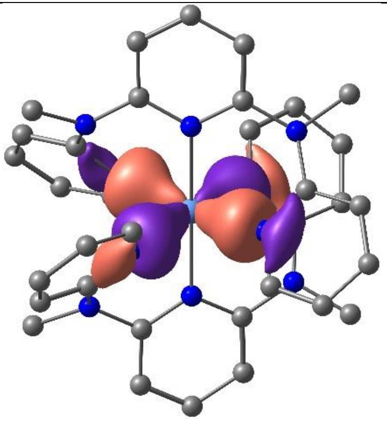	0.2236	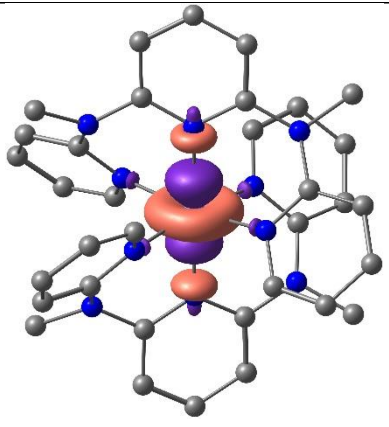 d_{z2}
–0.5369	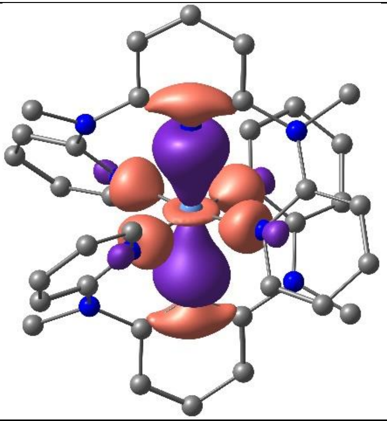	1.0137	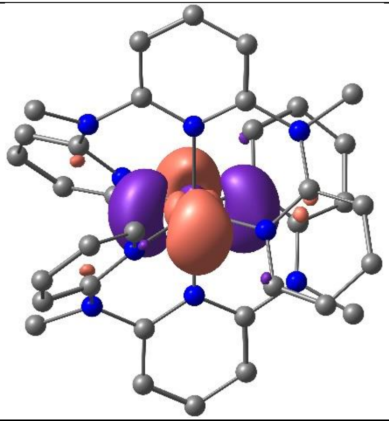
–0.0480	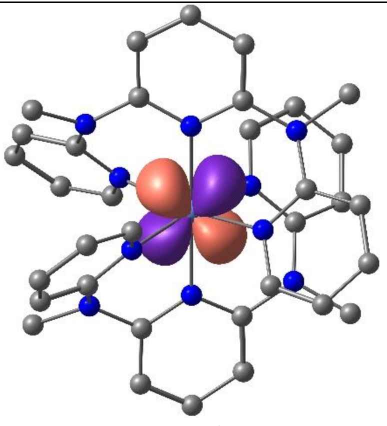 d_{xz} / d_{yz}	1.1190	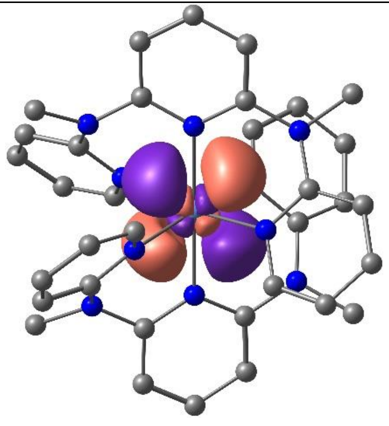

−0.0326	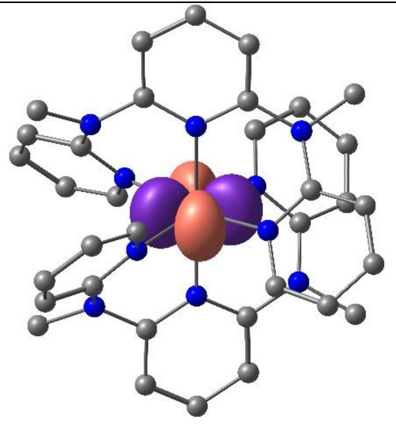 d_{xy}	1.0470	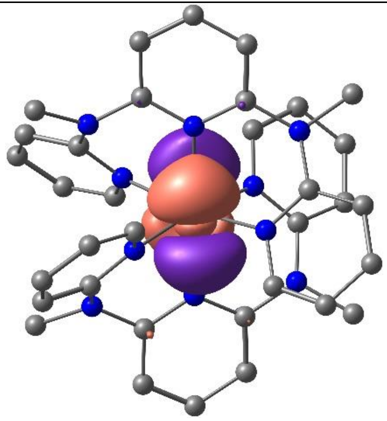
−0.0178	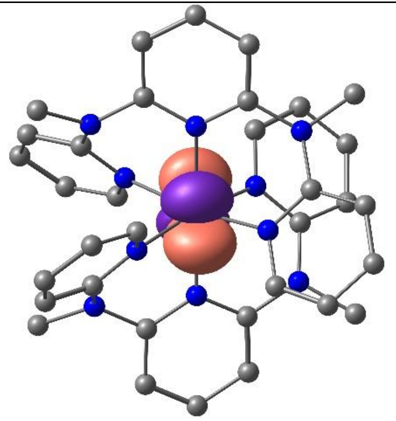 d_{xz} / d_{yz}	1.6061	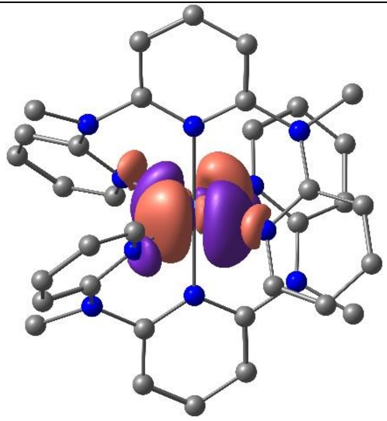
0.2122	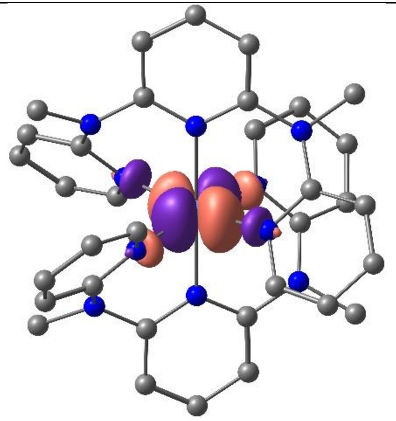 d_{x2-y2}	1.7641	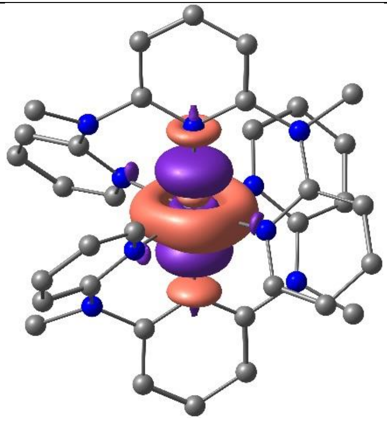

Table S5. Energies of ligand-field states with dominantly contributing orbital populations of $[Cr(ddpd)_2]^{2+}$ with S = 2 ground state obtained from CASSCF(8,12)-FIC-NEVPT2 calculations.

***E* / cm^{-1}**	**multiplicity**	**orbital populations $d_{xy}/d_{xz}/d_{yz}/d_{z2}/d_{x2-y2}$ (weight)**
0	5	11110 (0.98)
7411.6	3	12100 (0.93)
7491.8	3	21100 (0.63)
8732.3	3	11200 (0.63)
9415.3	5	11101 (0.98)
15533.9	1	12100 (0.64)
15689.2	1	21100 (0.59)
15871.3	3	11110 (0.80)
16706.8	3	21010(0.70)
16734.4	1	11200 (0.59)
16735.1	1	22000 (0.33)<br>02200 (0.31)
16753.9	3	20110 (0.57)
17466.2	3	12010 (0.37)<br>20110 (0.25)<br>10210 (0.24)
17588.6	1	20200 (0.49)<br>02200 (0.16)<br>11110 (0.13)
17721.0	5	11011 (0.57)<br>01111 (0.41)
18012.8	3	11110 (0.88)
19576.2	3	11110 (0.86)
19785.2	5	10111 (0.99)
20019.3	5	01111 (0.57)<br>11011 (0.41)
22179.2	1	11110 (0.34)<br>02200 (0.30)
22389.9	3	10210 (0.33)<br>10201 (0.22)<br>12001 (0.21)
23654.1	1	21010 (0.74)
24161.6	3	02101 (0.69)
24181.0	1	20110 (0.79)
25387.0	1	11110 (0.70)
27036.2	1	12010 (0.44)<br>10210 (0.29)
29973.5	1	10201 (0.31)<br>12001 (0.25)

Table S6. Energies of ligand-field states with dominantly contributing orbital populations of $[Cr(ddpd)_2]^{2+}$ with S = 1 ground state obtained from CASSCF(8,12)-FIC-NEVPT2 calculations.

energy / cm^{-1}	multiplicity	orbital populations (d_{xz}/d_{yz})/d_{xy}/(d_{xz}/d_{yz})/d_{x2-y2}/d_{z2} (weight)
0	3	12100 (0.94)
218.4	3	21100 (0.94)
1046.4	3	11200 (0.94)
1070.9	5	11110 (0.98)
1932.8	5	11101 (0.98)
8089.9	1	22000 (0.64)
8237.6	1	21100 (0.89)
9195.2	1	11200 (0.88)
9492.2	1	12100 (0.88)
10172.3	1	20200 (0.55)
15836.7	3	11110 (0.79)
16229.4	3	20110 (0.55)
16502.1	3	11101 (0.66)
16605.3	1	02200 (0.26)<br>20200 (0.20)<br>11110 (0.19)
16931.3	3	10210 (0.48)<br>20110 (0.35)
17093.0	3	12001 (0.55)
17099.9	3	11101 (0.80)
17242.2	3	11110 (0.72)
17997.0	3	02101 (0.48)<br>20110 (0.42)
18633.4	5	11011 (0.99)
19741.7	5	10111 (0.99)
20261.1	5	01111 (0.99)
23528.7	1	11110 (0.80)
23640.4	1	12001 (0.45)<br>20110 (0.35)
23735.5	1	21010 (0.43)<br>11101 (0.38)
24355.9	1	02101 (0.42)<br>10210 (0.34)
25173.0	1	12001 (0.43)<br>20110 (0.42)

Table S7. Cartesian coordinates of geometry optimized $[Cr(ddpd)_2]^{2+}$ (S = 2)

Cr	0.000000000	0.000000000	0.000000000
C	0.973797000	0.641472000	2.825511000
C	1.003457000	0.650228000	4.217180000
C	-0.000250000	0.004190000	4.914222000
C	-1.003045000	-0.644255000	4.218057000
C	-0.971766000	-0.639847000	2.826484000
N	0.001269000	-0.000082000	2.139938000
H	1.808316000	1.142148000	4.738454000
H	-1.808337000	-1.134934000	4.739871000
C	3.039371000	-0.780218000	-0.509945000
C	4.296198000	-0.323840000	-0.845964000
C	4.792289000	0.774552000	-0.146627000
C	4.032854000	1.356840000	0.851141000
C	2.766416000	0.821065000	1.135144000
N	2.282708000	-0.218635000	0.444573000
H	5.771912000	1.173493000	-0.371731000
H	2.591367000	-1.617592000	-1.029569000
H	4.863999000	-0.803325000	-1.629210000
H	4.428021000	2.188295000	1.409538000
C	-2.763358000	-0.825730000	1.136018000
C	-4.026622000	-1.367243000	0.848413000
C	-4.787005000	-0.786415000	-0.149495000
C	-4.294996000	0.315814000	-0.845698000
C	-3.040607000	0.776992000	-0.507115000
N	-2.282961000	0.217073000	0.447675000
H	-5.764199000	-1.189728000	-0.377374000
H	-4.418302000	-2.202765000	1.403226000
H	-4.863877000	0.794325000	-1.628759000
H	-2.595300000	1.617215000	-1.024507000
C	0.733927000	0.908145000	-2.737182000
C	0.759466000	0.927542000	-4.127370000
C	0.001346000	0.000496000	-4.819653000
C	-0.757690000	-0.926562000	-4.128423000
C	-0.734052000	-0.907181000	-2.738181000
N	-0.000525000	0.000503000	-2.060000000
H	1.332891000	1.670726000	-4.654919000
H	-1.330220000	-1.669921000	-4.656690000
C	-0.549983000	2.869729000	0.750273000
C	-0.295401000	4.215222000	0.884214000
C	0.633781000	4.792773000	0.022237000
C	1.243191000	4.018616000	-0.945701000
C	0.919632000	2.658680000	-1.033370000
N	0.059453000	2.099296000	-0.169146000
H	0.870106000	5.845509000	0.091693000
H	-1.244444000	2.356414000	1.397758000
H	-0.798847000	4.790315000	1.646333000
H	1.934514000	4.466400000	-1.639328000

C	-0.921258000	-2.657964000	-1.034973000
C	-1.246078000	-4.017539000	-0.946740000
C	-0.636556000	-4.792157000	0.020727000
C	0.293923000	-4.215434000	0.881829000
C	0.549245000	-2.870111000	0.747830000
N	-0.060270000	-2.099154000	-0.171146000
H	-0.873947000	-5.844632000	0.090569000
H	-1.939259000	-4.464295000	-1.639077000
H	0.797602000	-4.790926000	1.643489000
H	1.244778000	-2.357420000	1.394606000
N	1.494868000	1.845153000	-2.014173000
C	2.702333000	2.351799000	-2.673647000
H	2.492945000	3.141777000	-3.396980000
H	3.375550000	2.734209000	-1.912620000
H	3.191627000	1.524135000	-3.176630000
N	-1.495626000	-1.844593000	-2.016427000
C	-2.700954000	-2.352696000	-2.678914000
H	-2.488827000	-3.142037000	-3.402134000
H	-3.375363000	-2.736646000	-1.919729000
H	-3.190216000	-1.525487000	-3.182688000
N	1.962921000	1.366480000	2.133987000
N	-1.959461000	-1.367994000	2.136020000
C	2.408327000	2.616570000	2.755344000
H	3.266606000	2.476366000	3.415025000
H	2.673160000	3.323668000	1.973317000
H	1.585119000	3.034895000	3.323800000
C	-2.402996000	-2.617331000	2.760109000
H	-3.261444000	-2.476830000	3.419592000
H	-2.666770000	-3.326542000	1.979781000
H	-1.579226000	-3.033045000	3.329587000
H	-0.000870000	0.005943000	5.995257000
H	0.002120000	0.000458000	-5.900620000

Table S6. Cartesian coordinates of geometry optimized $[Cr(ddpd)_2]^{2+}$ (S = 1)

Cr	0.000000000	0.000000000	0.000000000
C	0.955267000	0.673265000	2.790410000
C	0.972258000	0.698867000	4.181419000
C	-0.001557000	0.001813000	4.873342000
C	-0.974738000	-0.696240000	4.181446000
C	-0.956510000	-0.672398000	2.790475000
N	-0.000401000	0.000125000	2.110916000
H	1.751704000	1.223479000	4.708027000
H	-1.754577000	-1.220280000	4.708056000
C	2.855550000	-0.648287000	-0.750315000
C	4.215416000	-0.470058000	-0.865184000
C	4.845632000	0.382391000	0.037137000
C	4.095814000	0.994952000	1.024688000
C	2.717359000	0.768874000	1.080001000
N	2.102098000	-0.011966000	0.171937000
H	5.912546000	0.549642000	-0.010180000
H	2.318258000	-1.294367000	-1.427682000
H	4.763554000	-0.984772000	-1.639962000
H	4.575624000	1.621720000	1.757864000
C	-2.717363000	-0.771320000	1.078724000
C	-4.095232000	-1.000421000	1.020505000
C	-4.844406000	-0.388559000	0.032043000
C	-4.214461000	0.466063000	-0.868368000
C	-2.855164000	0.646954000	-0.750994000
N	-2.102246000	0.011303000	0.172129000
H	-5.910853000	-0.558187000	-0.017426000
H	-4.575524000	-1.629130000	1.751690000
H	-4.762337000	0.980213000	-1.643697000
H	-2.317666000	1.294500000	-1.426824000
C	0.671111000	0.956670000	-2.790418000
C	0.694541000	0.975167000	-4.181462000
C	-0.001259000	0.000144000	-4.873182000
C	-0.696637000	-0.975047000	-4.181363000
C	-0.672070000	-0.956998000	-2.790254000
N	-0.000147000	-0.000324000	-2.110833000
H	1.216545000	1.756614000	-4.707943000
H	-1.219245000	-1.756231000	-4.707820000
C	-0.649203000	2.854748000	0.751033000
C	-0.468156000	4.213984000	0.868943000
C	0.387257000	4.844019000	-0.030693000
C	0.999207000	4.095094000	-1.019324000
C	0.769372000	2.717383000	-1.078363000
N	-0.013147000	2.101907000	-0.171888000
H	0.557675000	5.910327000	0.019666000
H	-1.297021000	2.317224000	1.426522000
H	-0.982782000	4.761578000	1.644143000
H	1.628156000	4.574815000	-1.750706000

C	-0.770807000	-2.717348000	-1.077632000
C	-1.001972000	-4.094833000	-1.017551000
C	-0.390412000	-4.843565000	-0.028605000
C	0.465644000	-4.213703000	0.870566000
C	0.647963000	-2.854722000	0.751737000
N	0.012612000	-2.102063000	-0.171850000
H	-0.561718000	-5.909701000	0.022461000
H	-1.631550000	-4.574489000	-1.748423000
H	0.979740000	-4.761258000	1.646147000
H	1.295873000	-2.317150000	1.427137000
N	1.371329000	1.939126000	-2.074328000
C	2.571805000	2.485199000	-2.713489000
H	2.353821000	3.269204000	-3.440892000
H	3.219040000	2.889005000	-1.939498000
H	3.095492000	1.674543000	-3.210924000
N	-1.372157000	-1.939380000	-2.074043000
C	-2.573383000	-2.484580000	-2.712491000
H	-2.356227000	-3.268801000	-3.439915000
H	-3.220385000	-2.888035000	-1.938124000
H	-3.096843000	-1.673457000	-3.209358000
N	1.937989000	1.372799000	2.074158000
N	-1.938599000	-1.373284000	2.074366000
C	2.482834000	2.574946000	2.711114000
H	3.266300000	2.359092000	3.439927000
H	2.887387000	3.220581000	1.936136000
H	1.671528000	3.099657000	3.206368000
C	-2.482443000	-2.575503000	2.712056000
H	-3.265571000	-2.360244000	3.441418000
H	-2.886882000	-3.221787000	1.937621000
H	-1.670668000	-3.099439000	3.207387000
H	-0.002068000	0.002531000	5.954449000
H	-0.001655000	0.000362000	-5.954292000

References

[S1] W. W. Schoeller, J. Niemann, *J. Am. Chem. Soc.* **1986**, *108*, 22–26.

[S2] A. Breivogel, C. Förster, K. Heinze, *Inorg. Chem.* **2010**, *49*, 7052–7056.

[S3] R. A. Heintz, J. A. Smith, P. S. Szalay, A. Weisgerber, K. R. Dunbar in *Inorg. Synth.* **2002**, *33*, 75–83, (Ed. D. Coucouvanis), John Wiley & Sons, Inc.

[S4] a) P. Neugebauer, D. Bloos, R. Marx, P. Lutz, M. Kern, D. Aguila, J. Vaverka, O. Laguta, C. Dietrich, R. Clerac, J. van Slageren, *Phys. Chem. Chem. Phys.* **2018**, *20*, 15528–15534; b) S. Lenz, H. Bamberger, P. P. Hallmen, Y. Thiebes, S. Otto, K. Heinze, J. van Slageren, *Phys. Chem. Chem. Phys.* **2019**, *21*, 6976–6983.

[S5] S. Stoll, A. Schweiger, *J. Magn. Reson.* **2006**, *178*, 42–55.

[S6] M. Zimmer, F. Rupp, P. Singer, F. Walz, F. Breher, W. Klopper, R. Diller, M. Gerhards, *Phys. Chem. Chem. Phys.* **2015**, *17*, 14138–14144.

[S7] F. Bäppler, M. Zimmer, F. Dietrich, M. Gruppe, M. Wallesch, D. Volz, S. Bräse, M. Gerhards, R. Diller, *Phys. Chem. Chem. .Phys.* **2017**, *19*, 29438–29448.

[S8] M. Zimmer, F. Dietrich, D. Volz, S. Bräse, M. Gerhards, *ChemPhysChem* **2017**, *18*, 3023–3029.

[S9] STOE & Cie, X-Area, STOE & Cie, Darmstadt, Germany.

[S10] R. H. Blessing, *Acta Crystallogr. Sect. A* **1995**, *51*, 33–38.

[S11] A. L. Spek, *Acta Crystallogr. Sect. D* **2009**, *65*, 148–55.

[S12] a) G. M. Sheldrick, *Acta Crystallogr. Sect. A* **2015**, *71*, 3–8; b) G. M. Sheldrick, SHELXL-2014/7, University of Göttingen, Göttingen, Germany, **2014**.

[S13] C. B. Hübsche, G. M. Sheldrick, B. Dittrich, *J. Appl. Cryst.* **2011**, *44*, 1281–1284.

[S14] F. Neese, *WIREs Comput. Mol. Sci.* **2012**, *2*, 73–78.

[S15] F. Neese, F. Wennmohs, A. Hansen, U. Becker, *Chem. Phys.* **2009**, *356*, 98–109.

[S16] R. Izsák, F. Neese, *J. Chem. Phys.* **2011**, *135*, 144105.

[S17] A. D. Becke, *J. Chem. Phys.* **1993**, *98*, 5648–5652.

[S18] F. Weigend, R. Ahlrichs, *Phys. Chem. Chem. Phys.* **2005**, *7*, 3297–3305.

[S19] F. Weigend, *Phys. Chem. Chem. Phys.* **2006**, *8*, 1057–1065.

[S20] D. A. Pantazis, X.-Y. Chen, C. R. Landis, F. Neese, *J. Chem. Theory Comput.* **2008**, *4*, 908–919.

[S21] E. van Lenthe, E. J. Baerends, J. G. Snijders, *J. Chem. Phys.* **1993**, *99*, 4597–4610.

[S22] S. Grimme, J. Antony, S. Ehrlich, H. Krieg, *J. Chem. Phys.* **2010**, *132*, 154104.

[S23] S. Grimme, S. Ehrlich, L. Goerigk, *J. Comput. Chem.* **2011**, *32*, 1456–1465.

[S24] V. Barone, M. Cossi, *J. Phys. Chem. A* **1998**, *102*, 1995–2001.

[S25] C. Angeli, R. Cimiraglia, S. Evangelisti, T. Leininger, J.-P. Malrieu, *J. Chem. Phys.* **2001**, *114*, 10252−10264.

[S26] C. Angeli, R. Cimiraglia, *Theor. Chem. Acc.* **2002**, *107*, 313−317.

[S27] K. Pierloot, *Int. J. Quantum Chem.* **2011**, *111*, 3291–3301.
